# *Aspergillus fumigatus* MADS-Box Transcription Factor *rlmA* Is Required for Regulation of the Cell Wall Integrity and Virulence

**DOI:** 10.1534/g3.116.031112

**Published:** 2016-07-28

**Authors:** Marina Campos Rocha, João Henrique Tadini Marilhano Fabri, Krissia Franco de Godoy, Patrícia Alves de Castro, Juliana Issa Hori, Anderson Ferreira da Cunha, Mark Arentshorst, Arthur F. J. Ram, Cees A. M. J. J. van den Hondel, Gustavo Henrique Goldman, Iran Malavazi

**Affiliations:** * Departamento de Genética e Evolução, Centro de Ciências Biológicas e da Saúde, Universidade Federal de São Carlos, 13565 São Carlos, São Paulo, Brazil; † Faculdade de Ciências Farmacêuticas de Ribeirão Preto, Universidade de São Paulo, Brazil; ‡ Departamento de Farmacologia, Faculdade de Medicina de Ribeirão Preto, Universidade de São Paulo, 14049 Ribeirão Preto, São Paulo, Brazil; § Molecular Microbiology and Biotechnology, Institute of Biology, Leiden University, 2333 Leiden, The Netherlands

**Keywords:** *Aspergillus fumigatus*, *rlmA*, cell wall integrity, virulence, *mpkA*

## Abstract

The Cell Wall Integrity (CWI) pathway is the primary signaling cascade that controls the *de novo* synthesis of the fungal cell wall, and in *Saccharomyces cerevisiae* this event is highly dependent on the *RLM1* transcription factor. Here, we investigated the function of RlmA in the fungal pathogen *Aspergillus fumigatus*. We show that the Δ*rlmA* strain exhibits an altered cell wall organization in addition to defects related to vegetative growth and tolerance to cell wall-perturbing agents. A genetic analysis indicated that *rlmA* is positioned downstream of the *pkcA* and *mpkA* genes in the CWI pathway. As a consequence, *rlmA* loss-of-function leads to the altered expression of genes encoding cell wall-related proteins. RlmA positively regulates the phosphorylation of MpkA and is induced at both protein and transcriptional levels during cell wall stress. The *rlmA* was also involved in tolerance to oxidative damage and transcriptional regulation of genes related to oxidative stress adaptation. Moreover, the Δ*rlmA* strain had attenuated virulence in a neutropenic murine model of invasive pulmonary aspergillosis. Our results suggest that RlmA functions as a transcription factor in the *A. fumigatus* CWI pathway, acting downstream of PkcA-MpkA signaling and contributing to the virulence of this fungus.

*Aspergillus fumigatus* is a saprophyte filamentous fungus that is ubiquitously distributed around the world, which plays an important role in carbon and nitrogen recycling in soil (Tekaia and Latge 2005). This organism is also an aggressive opportunistic human pathogen that causes systemic infections in immunocompromised individuals (Steinbach 2008; Segal 2009). Among the range of diseases caused by this fungus, invasive pulmonary aspergillosis (IPA) is the life-threatening form of infection and is associated with high mortality rates of 50–90% in the currently increasing population of immunocompromised patients (Dagenais and Keller 2009; Kousha *et al.* 2011; Brown *et al.* 2012, 2014). The conidia from this fungus are highly abundant and mostly disperse in the air. They can be inhaled by the mammalian host and then reach and colonize the lungs (Kwon-Chung and Sugui 2013). IPA is a multifactorial disease, given that several *A. fumigatus* virulence determinants and phenotypic traits support the capacity of this organism to cause disease in immunocompromised hosts (Krappmann 2008). Some examples of these traits are nutritional versatility, thermotolerance, and the secretion of secondary metabolites including gliotoxin and siderophores (Brown and Goldman 2016). These features ultimately allow this fungus to adapt and colonize the host’s environment and evade its defense mechanisms (Brakhage and Langfelder 2002; Araujo and Rodrigues 2004; Bhabhra and Askew 2005; Tekaia and Latge 2005; Sugui *et al.* 2007; Schrettl and Haas 2011; Haas 2014; Brown and Goldman 2016).

The fungal cell wall has been shown to perform multiple roles in virulence because *A. fumigatus* mutants that have deficiencies in cell wall integrity (CWI) have attenuated virulence (Mouyna *et al.* 2005; Beauvais *et al.* 2013; Bom *et al.* 2015; Winkelstroter *et al.* 2015; Bruder Nascimento *et al.* 2016). Fungal cell survival is highly dependent on the organization, composition, and function of the cell wall component. This structure is essential for providing an adequate cell shape and integrity to fungal morphotypes, preventing cell lysis. In addition, this structure plays a role in cell-to-cell adhesion and in the prevention of nonself recognition by the host immune system (Gastebois *et al.* 2009; Dirr *et al.* 2010; Latge 2010). As a rigid but dynamic protective barrier, this structure is under constant biosynthesis and remodeling as forced by the inherent processes involved in fungal growth and reproduction (Klis *et al.* 2006; Levin 2011; Piccirillo *et al.* 2015) or by the multiple environmental challenges that are sensed by the invading pathogen during infection.

The CWI pathway is a signal transduction cascade that maintains the integrity of the cell wall. The CWI pathway is conserved among fungi and has been studied in many human fungal pathogens including *A. fumigatus* (Valiante *et al.* 2008, 2009; Fuchs and Mylonakis 2009; Dirr *et al.* 2010; Dichtl *et al.* 2012, 2016; Samantaray *et al.* 2013; Rocha *et al.* 2015). In *Saccharomyces cerevisiae*, the CWI pathway is the primary signaling cascade that is activated in response to cell wall stress. Cell wall damage is initially sensed by mechanosensors that are located on the plasma membrane. The signal is transmitted to Pkc1 (the apical kinase in the CWI pathway) through the activity of the small Rho GTPase Rho1 and two guanine nucleotide exchange factors (GEFs) called Rom1-2 (reviewed in Levin 2011). The Pkc1 module of the circuit comprises a downstream mitogen-activated protein kinase (MAPK)-signaling component, which amplifies the CWI signal. The MAPK module of the CWI pathway is a linear three-component kinase consisting of Bck1, two redundant proteins (Mkk1 and Mkk2), and the final kinase known as Mpk1 (Slt2). These proteins are sequentially phosphorylated and activated via the Pkc1-mediated upcoming signal. The activated Mpk1 controls the function of two transcription factors, namely SBF (Swi4/Swi6) and Rlm1, which are responsible for regulating the expression of genes involved in cell cycle control and cell wall biosynthesis, respectively (Watanabe *et al.* 1995, 1997; Dodou and Treisman 1997; Gustin *et al.* 1998; Jung *et al.* 2002). However, there is also some interplay between these two transcription factors in CWI signaling (Madden *et al.* 1997; Baetz *et al.* 2001).

Rlm1 is a member of the MADS (Mcm1-Agamous-Deficiens-Serum Response Factor)-box transcription factor family, which controls diverse developmental processes, especially in plants (Smaczniak *et al.* 2012). Yeast and fungal RLM1-related genes are type II MADS-box transcription factors, which also include the human MEF2 (Myocyte-Enhancer-Factor 2) gene (West *et al.* 1997; Becker and Theissen 2003). One of the direct consequences of yeast Rlm1 activation through Mpk1 phosphorylation in response to cell wall damage is the transcriptional regulation of several genes related to cell wall metabolism (Watanabe *et al.* 1997; Jung and Levin 1999; Roberts *et al.* 2000; Garcia *et al.* 2004).

Although the components of the PkcA-MpkA pathway have been functionally characterized in *A. fumigatus* (Valiante *et al.* 2008, 2009; Dirr *et al.* 2010; Rocha *et al.* 2015; Dichtl *et al.* 2016), the participation of the putative downstream *RLM1* ortholog *rlmA* in the CWI pathway has not been elucidated. In addition, several authors have suggested that the *A. fumigatus* PkcA-MpkA circuit is not the only one that is responsible for promoting the CWI pathway (Fujioka *et al.* 2007; Rocha *et al.* 2015; Valiante *et al.* 2015; Bruder Nascimento *et al.* 2016). Thus, in the present study, we aimed to investigate the involvement of the *A. fumigatus rlmA* gene in the CWI pathway and virulence. We showed that RlmA plays a central role in responses to cell wall stress and positively regulates MpkA phosphorylation. We propose that RlmA is acting downstream of the other components of the canonical *A. fumigatus* CWI pathway, namely PkcA and MpkA, and that this signaling circuit is required for the transcriptional activation of genes that encode cell wall biosynthesis enzymes. The role of *rlmA* as a transcription factor in response to cell wall stress has a dramatic impact on *A. fumigatus* virulence and pathogenicity, given that the Δ*rlmA* strain showed attenuated virulence in a murine model of invasive pulmonary aspergillosis.

## Materials and Methods

### Strains and culture conditions

The *A. fumigatus* strains used in this study are described in Supplemental Material, Table S1. All the strains were maintained in complete medium [YG; glucose 2% (w/w), 0.5% yeast extract (w/w), and 1 × trace elements)] or minimal medium [MM; glucose 1% (w/w), 1 × high nitrate salt solution, and 1 × trace elements, pH 6.5]. The composition of the trace elements and high nitrate salt solution were described previously (Kafer 1977). For solid media, 2% agar (w/w) was added. To grow the *akuB*^KU80^ *pyrG*^-^ strain, the media was supplemented with 1.2 g/L of uridine and uracil. MM+sorbitol had the same composition as MM, but contained D-sorbitol (1.2 M). The growth rate was determined at different temperatures by spotting 1 × 10^4^ conidia into the center of a 90 mm Petri dish containing 20 ml of solid YG medium. The diameter was scored at 24 hr intervals.

To assess the germination kinetics of the Δ*rlmA* mutant strain, 1 × 10^6^ conidia of each strain were inoculated onto glass coverslips that were placed in a 35 mm Petri dish containing 2 ml of YG medium, which was incubated at 37° or 45° for 2, 4, 6, and 8 hr. After incubation, the coverslips with adherent germlings were transferred to fixative solution [PBS 1 ×, DMSO 5% (v/v), and formaldehyde 3.7% (v/v)] for 10 min at room temperature. The coverslips were rinsed briefly with PBS buffer, mounted, and visualized under a bright field microscope. A conidiospore was counted as germinated if it possessed a germ tube, which is readily detectable as a small protuberance on the spherical spore surface. Two hundred conidia were counted in each experiment.

To induce cell wall stress, 1 × 10^7^ conidia from wild-type and mutant strains were incubated in 50 ml of liquid YG for 16 hr or MM for 24 hr. To grow the Δ*mpkA* strain, four times more conidia were used to achieve equal glucose consumption, as described previously (Jain *et al.* 2011). Following incubation, 300 µg/ml of Congo Red (CR) was added to the cultures, and they were incubated for an additional 15, 30, and 60 min. The control was left untreated. To induce oxidative stress, the same procedures were used except that hydrogen peroxide (5 mM) was added to the cultures. Mycelia from each time point, for both pre- and post-CR or H_2_O_2_ exposure, were collected via vacuum filtration, immediately frozen in liquid nitrogen, and stored at −80° until used for either RNA or protein extractions.

### Deletion and reconstitution of the rlmA gene and the construction of the rlmA::gfp strain

The gene replacement cassette was constructed by *in vivo* recombination in *S. cerevisiae* as reported in (Malavazi and Goldman 2012). In brief, two fragments encompassing the *rlmA* (Afu3g08520 ) gene were PCR-amplified from the genomic DNA of the CEA17 strain according to Figure S1. The primers used are listed in Table S2. The 5′ and 3′ *rlmA* flanking sections contained a short sequence that was homologous to the multiple cloning site of the pRS426 plasmid (the small letters indicated in Table S2). The *pyrG* gene that was inserted into the gene replacement cassette was amplified from pCDA21 plasmid (Chaveroche *et al.* 2000) and used to generate a prototrophy marker in the mutant strain. The deletion cassette was generated by transforming the three independent fragments along with the *Bam*HI-*Eco*RI-cut pRS426, into the *S. cerevisiae* FGSC 9721 (FY834) strain by the lithium acetate method described in Malavazi and Goldman (2012). Genomic DNA that was extracted from the *S. cerevisiae* transformant cells was used to transform *Escherichia coli* chemocompetent DH5α cells to rescue the recombined pRS426 plasmid harboring the gene replacement cassette. The isolated plasmid was used as a template to PCR-amplify the cassette with the outermost primers (5F and 3R) indicated in Figure S1A. All the PCR amplifications were performed using Phusion Hot Start II High-Fidelity DNA Polymerase (Thermo Scientific). The gene replacement cassette was transformed into the *A. fumigatus* Δ*akuB*^KU80^ wild-type strain by using the polyethylene glycol-mediated protoplast technique according to the previously described procedures (Osmani *et al.* 1987; Malavazi and Goldman 2012).

To complement the Δ*rlmA* strain, the *rlmA* gene plus the two 1.0 kb flanking regions were PCR-amplified using the genomic DNA from the CEA17 strain as a template and the primers rlmA 1000 FW and rlmA 1000 REV (Table S2). Protoplasts from the Δ*rlmA* strain were transformed with the gel-purified PCR product and plated onto media containing 300 µg/ml of CR. Several revertants, which were able to grow under these conditions, were further analyzed by PCR, with the following primer sets: rlmA 600 ups/pyrG REV and rlmA ST SC 5F/rlmA ORF REV (Figure S1C upper and lower panel). The complemented strains were also tested for complementing phenotypes, and they yielded the same results. One of these strain was chosen and named as Δ*rlmA*::*rlmA^+^*.

To construct the double mutants of the CWI pathway genes, we generated a Δ*rlmA pyrG*^-^ strain which was obtained by the spontaneous loss of the *pyrG* prototrophy marker upon 5-FOA treatment. The substitution *pkcA*^G579R^ cassette was amplified from the pRS426 plasmid containing the recombined cassette as described previously (Rocha *et al.* 2015) and transformed into the Δ*rlmA pyrG*^-^ recipient strain to obtain the double mutant Δ*rlmA pkcA*^G579R^. The *pkcA* gene replacement in this mutant was checked by PCR with the primer set cpkcA FW and pyrG REV yielding a 6.7 kb band (Figure S1D). To generate the double mutants Δ*mpkA* Δ*rlmA* and Δ*mpkA pkcA*^G579R^, the *mpkA* deletion cassette was amplified from the genomic DNA of the Δ*mpkA* strain using primers MpkA_5′_For and MpkA_3′_Rev and transformed into both the Δ*rlmA* and *pkcA*^G579R^ strains. The *mpkA* deletion cassette contains the *ptrA* gene as a selectable marker (Valiante *et al.* 2009). The *mpkA* replacement in these double mutants was checked by using the primers mpkA 500 ups and MpkA_3′_Rev (Table S2), which can be used to discriminate the *mpkA* deletion from the wild-type *mpkA* locus by yielding a 5.5 kb or a 5.0 kb band, respectively (Figure S1, E and F, respectively).

To generate the *rlmA*::*gfp* strain, a substitution cassette was constructed in which an *rlmA* genomic sequence without a stop codon (1929 bp) was cloned in-frame with the green fluorescent protein (GFP) gene in a C-terminal fusion. The *rlmA* ORF was amplified by primers rlmA ST SC FW and rlmA ORF REV (Figure S1G). A four-residue linker consisting of Gly-Thr-Arg-Gly was inserted between the C-terminus of the *rlmA* and the start codon of GFP, as described elsewhere (Teepe *et al.* 2007). The GFP gene (726 bp) was PCR-amplified from the pMCB17apx plasmid by using the primers Spacer GFP FW and GFP REV pyrG. The *pyrG* (1911 bp) gene was also used in this cassette as a marker for prototrophy. The amplification of the *rlmA* 3′ UTR was performed with the same primers that were used in the construction of the deletion cassette, *i.e.*, Afu3g08520 3F and Afu3g08520 3R (Table S2). A *S. cerevisiae in vivo* recombination assay was performed as previously described. The PCR-amplified *rlmA*::*gfp* cassette was transformed into the *A. fumigatus* wild-type strain. Transformants were carefully tested by PCR with primers rlmA 5F and GFP REV pyrG to confirm the *rlmA* locus replacement (Figure S1H). As an additional control procedure for validating the *rlmA*::*gfp* strain, the substitution cassette *rlmA*::*gfp* was also transformed into a Δ*rlmA pyrG*- strain. All the revertants behaved like the wild-type in the presence of CR and CFW (data not shown).

### DNA manipulation and Southern blot analysis

Southern blot analysis was used to show that the deletion *rlmA* cassette integrated homologously at the targeted *A. fumigatus rlmA* locus. Genomic DNA from *A. fumigatus* was extracted by grinding frozen mycelia in liquid nitrogen and then the genomic DNA was extracted as previously described (Malavazi and Goldman 2012). For Southern blot analysis, *Bam*HI- and *Xho*I-restricted chromosomal DNA fragments were separated on a 1% agarose gel and blotted onto Hybond N^+^ nylon membranes (GE Healthcare). Probe labeling for detection was performed using [α-^32^P]dCTP with the Random Primers DNA Labeling System (Life Technologies). Labeled membranes were exposed to X-ray films, which were scanned for image processing.

### Susceptibility assay for cell wall, oxidative, and endoplasmic reticulum stress

To monitor growth under cell wall stress, serial dilutions of conidia were spotted onto agar plates that were supplemented with varying concentrations of caffeine (CAFF), calcofluor white (CFW), congo red (CR), anidulafungin, (AF), caspofungin (CASP), sodium dodecyl sulfate (SDS), fluconazole (FLUC), and ethylenediaminetetraacetic acid (EDTA), and grown for 48 hr. Alternatively, to assess their sensitivity to nikkomycin Z, 1 × 10^5^ conidia of each strain were grown on 1 ml of solid media in 24-well plates. Endoplasmic reticulum (ER) stress in the presence of DTT (dithiothreitol) was likewise tested in liquid YG culture, and brefeldin A (BFA) and tunicamycin (TM) were tested in solid media, as previously described for *A. fumigatus* (Richie *et al.* 2009). The plates were incubated for 2–3 d at 37°, and the extent of vegetative growth was used as a relative indicator of sensitivity. For the experiments on solid MM supplemented with 1.2% sorbitol, serial dilutions of conidia ranging from 1 × 10^6^ to 1 × 10^3^ were spotted onto agar plates. To evaluate the oxidative stress tolerance, 1 × 10^5^ conidia were inoculated into 24-well plates containing 1 ml of liquid MM and varying concentrations of menadione, paraquat, or T-butyl hydroperoxide. Sensitivity to the oxidative damage generated by H_2_O_2_ was tested by inhibition zone assay on MM agar plates, as described by (Valiante *et al.* 2008). Antifungal susceptibility by E-test diffusion assay was performed essentially as described by Richie *et al.* (2009).

### Construction of PagsA::mluc reporter and luciferase activity assay

A schematic overview of the *agsA*::*mluc* reporter construct (plasmid pNB04) is given in Figure S2. The details on the construction of the plasmid will be published elsewhere (C. A. M. J. J. van den Hondel *et al.*, unpublished results). The plasmid contains the 5′ and 3′ regions of *A. fumigatus pyrG* gene for targeting the *pyrG* locus. Upstream of the luciferase reporter gene (*mluc*), the *A. niger agsA* promoter region is present containing three RlmA recognition sites (3 × Rlm-box). Downstream of the *mluc* gene, the termination region of the *A. nidulans trpC* gene is present. The bacterial hygromycin resistance gene is surrounded by *the A. nidulans gpdA* promoter and termination regions. The *Not*I-*Kpn*I DNA fragment of pNB04 (P*agsA*::*mluc* cassette) was introduced into *A. fumigatus* wild-type (Δ*akuB*^KU80^) and Δ*rlmA* strains. Fourteen hygromycin-resistant transformants for each strain were purified two times and analyzed for the correct replacement of the *A. fumigatus pyrG* gene by the P*agsA*::*mluc* cassette by PCR and Southern blot analysis (results not shown). The resulting strains were named as MAF 6.6 (wt; P*agsA*::*mluc*) and MAF 8.1(Δ*rlmA*; P*agsA*::*mluc*).

The luciferase activity assay described previously (Meyer *et al.* 2011) has been slightly modified (Arentshorst *et al.* 2012). Briefly, 50 μl of 2 × MM with 0.006% yeast extract (w/v) and 50 μl spore suspension (3 × 10^5^ conidia/ml) was pipetted together (in triplicate) in a well of a white, clear bottomed, 96-well plate (Greiner Bio-one, ref 655095) and incubated for 5 hr at 37°. Thereafter, 50 μl of 2 × MM with 0.006% yeast extract (w/v), 26 μl deionized water (MQ), 4 μl 25 mM luciferin (Promega, E1605), and 20 μl of different concentrations of freshly dissolved CFW in MQ were added and subsequently incubated at 30° in the EnSpire MultiplateReader (Perkin) with continuous measuring of luciferase luminescence and OD_600_.

### RNA extraction, cDNA preparation, and qRT-PCR procedures

Mycelia that were obtained according to the description for each qRT-PCR experiment were disrupted by grinding them in liquid nitrogen with a pestle and mortar. The total RNA was extracted with TRIzol reagent (Thermo Scientific) according to the manufacturer’s protocol. The samples were treated with Turbo DNase I (Ambion Thermo Scientific). The DNAse treatment was validated by real time PCR using *A. fumigatus* β-tubulin (*tubA*) primers with the DNAse-treated RNA as the template in the reactions. The quality of DNAse-treated RNA was confirmed with a denaturing agarose gel (2.2 M formaldehyde; 1.2% (w/v) agarose) stained with ethidium bromide. The RNA concentration and quality were measured with a nanophotometer (NanoVue, GE HealthCare). The RNA integrity was assessed with a 2100 Bioanalyzer (Agilent Technologies). A 2 µg quantity of DNAse-treated total RNA from each *A. fumigatus* strain was reverse-transcribed with a High Capacity cDNA Reverse Transcription kit (Thermo Scientific) using oligo dTV and random primers blend. qRT-PCR was conducted with a Power Sybr Green PCR Master Mix (Thermo Scientific). The primers for the individual genes were designed using Primer Express 3.0 software (Life Technologies) and are listed in Table S3. qRT-PCR was performed in duplicate with three independent biological samples in a StepOne Plus Real Time PCR System (Thermo Scientific). The concentration of each primer pair was optimized prior to the efficiency curve reaction. Only primers with amplification efficiency ranging from 95–105% were used, according to reference (Bustin *et al.* 2009). Nontemplate controls (NTC) were used to confirm the elimination of contaminating DNA in every run. A melt curve analysis was performed after the PCR was complete to confirm the absence of nonspecific amplification products. The fold change in mRNA abundance was calculated using 2^−ΔΔCt^ (Livak and Schmittgen 2001) and all the values were normalized to the expression of the *A. fumigatus* β-tubulin (*tubA)*, which encodes the β-2 tubulin subunit (Afu1g10910) gene.

### Protoplast counting

To assess the ability of the Δ*rlmA* strain to generate protoplasts in a lytic cocktail containing cell wall-degrading enzymes under standard conditions, 2 × 10^6^ conidia from each strain were inoculated in 50 ml of liquid YG and incubated for 16 hr at 37° (180 rpm). The cells were washed twice with sterile MilliQ water and 100 mg of mycelium wet weight was incubated in 50 ml of an osmotic stabilized protoplasting solution [(0.4 M ammonium sulfate, 50 mM citric acid pH 6.0, yeast extract 0.5% (w/v), and sucrose 1% (w/v)] according to (Malavazi and Goldman 2012) containing 0.3% Lallzyme MMX as the enzymatic cocktail and 400 mg of BSA at 30° (90 rpm). The protoplasts yield was analyzed using a Neubauer chamber after incubating for 0, 4, and 6 hr.

### Staining for dectin-1 and chitin

Staining was performed as previously described (Walker *et al.* 2008; Winkelstroter *et al.* 2015). In brief, *A. fumigatus* conidia were grown for 8 hr at 37° in liquid MM, UV-irradiated, blocked in blocking solution (goat serum 2%, BSA 1%, 0.1% Triton X-100, 0.05% Tween 20, 0.05% NaF, and 0.01 M PBS) for 1 hr at room temperature, and stained with conditioned medium containing 1 µg/ml of s-dectin-hFc (Invivogen) followed by DyLight 594-conjugated, goat anti-human IgG1 (Graham *et al.* 2006). For chitin staining, UV-irradiated germlings were treated with CFW 2 μg/ml for 5 min. After they were washed, the stained cells were visualized under identical imaging conditions for a parallel comparison with a Zeiss Observer Z1 fluorescence microscope. The staining was quantified as the average amount of staining relative to the total fungal area using ImageJ software (Schneider *et al.* 2012). One hundred cells were analyzed in each independent experiment (n = 3).

### Biofilm formation assay

The quantification of the initial stages of biofilm formation in *A. fumigatus* was performed as described by Gravelat *et al.* (2010). In brief, 2 × 10^4^ conidia were inoculated into 200 µL of YG media in 96-well polystyrene plates and allowed to grow for 24 hr at 37°. Following the incubation, the media was removed and the adhered mycelia were washed four times with sterile PBS. A 150 µl volume of a 0.5% (w/v) crystal violet solution was added to each well for 5 min to stain the residual mycelia. Excess stain was gently removed under running water. The residual biofilm was destained with 200 µl of 95% ethanol per well, overnight at room temperature. The biofilm density was measured by determining the absorbance of the destaining solution at 570 nm (BioRad).

### Transmission electron microscopy analysis

Wild-type, Δ*rlmA*, and complementing strains were gown in liquid YG medium during 24 hr. Cells were processed essentially as described previously (Bom *et al.* 2015) with modifications. Briefly, the mycelia were fixed in 0.1 M sodium phosphate buffer (pH 7.4) containing 2.5% (v/v) of glutaraldehyde for 24 hr at 4°. Samples were encapsulated in agar (2% w/v) and subjected to fixation (1% OsO_4_), contrasting (1% uranyl acetate), ethanol dehydration, and a two-step infiltration process with propylene oxide/EMbed 812 (Electron Microscopy Sciences) of 16 and 3 hr at room temperature. Additional infiltration was provided under vacuum at room temperature before embedding in BEEM capsules (Electron Microscopy Sciences) and polymerizing at 60° for 72 hr. Semithin (0.5 µm) survey sections were stained with toluidine blue to identify the areas of best cell density. Ultrathin sections (60 nm) were prepared and stained with uranyl acetate (1%) and lead citrate (2%). Transmission electron microscopy (TEM) images were obtained using a Philips CM-120 electron microscope at an acceleration voltage of 120 kV using a MegaView3 camera and iTEM 5.0 software (Olympus Soft Imaging Solutions GmbH). Cell wall thicknesses of 50 sections of different germlings were measured using magnification of 66,000 × and ITEM 5.0 software analysis.

### Protein extraction and immunoblotting analysis of MpkA and RlmA

The strains exposed to cell wall stress induced by CR were used to assess the phosphorylation status of MpkA and the total RlmA abundance. For protein extraction, 500 µl of lysis buffer was added to the ground mycelia as described previously (Rocha *et al.* 2015). The supernatants were collected and the protein concentrations were determined according to the Lowry method modified by Hartree (1972). A 50 µg quantity of protein from each sample was resolved in a 12% (w/v) SDS-PAGE gel and transferred to polyvinylidene difluoride (PVDF) membranes (BioRad). The phosphorylation of MpkA was examined using anti-phospho p44/42 and the total amount of MpkA was detected by using the anti p44/42 MAPK antibody (9101 and 9107, respectively; Cell Signaling Technologies) according to the manufacturer’s instructions. Anti γ-tubulin (yN-20; Santa Cruz Biotechnology) was used as the loading control in these experiments. Anti γ-tubulin antibodies were detected with peroxidase (HRP)-conjugated secondary antibody (sc-2020; Santa Cruz Biotechnology). The incubations were performed as described previously (Rocha *et al.* 2015).

To detect the total amount of RlmA, the RlmA::GFP fusion was detected by the anti GFP antibody (Sigma G1544) at a 1:1000 dilution in TBST containing 3% skimmed milk (predicted molecular weight: 91.590 kDa). Incubation was performed at 4° for 16 hr. The primary antibody was detected with an HRP-conjugated secondary antibody raised in rabbits (A0545; Sigma).

All the primary antibody detections here were performed at room temperature with the specified secondary antibody in TBST buffer for a 2 hr incubation. Chemoluminescent detection was performed by using an ECL Prime Western Blot detection kit (GE HealthCare). Images were generated by exposing the PVDF membranes to the ChemiDoc XRS gel imaging system (BioRad). The images were subjected to densitometric analysis in ImageJ software (Schneider *et al.* 2012).

### BMDMs preparation, phagocytosis index, and the determination of TNF-α levels

For the cytokine quantification and phagocytosis index determination, Bone Marrow-Derived Macrophages (BMDMs) from C57BL/6 mice were prepared as previously described (Marim *et al.* 2010). In brief, bone marrow cells from the femurs of adult mice were cultured for 6 d in RPMI 1640 containing 20% fetal bovine serum (FBS) and 30% L-929 cell conditioned media (LCCM).

A phagocytic assay was performed according to Mech *et al.* (2011). In brief, 2 × 10^4^ macrophages were incubated with 1 ml of RPMI-FBS at 37° with 5% CO_2_ for 1 hr in a 24-well plate containing one 15 mm diameter coverslip per well. Next, the cells were washed with 1 ml of assay medium to remove nonadherent cells. In each well, 1 ml of RPMI-FBS containing 1 × 10^5^ conidia (1:5 macrophage/conidia ratio) was added. The samples were incubated at 37° and 5% CO_2_ for 80 min, and then the supernatant was removed and 500 μl of 3.7% formaldehyde-PBS was added. After 15 min, the samples were washed with 1 ml of ultrapure water and incubated for an additional 20 min with 495 μl of water and 5 μl of CFW (10 mg/ml). The samples were washed and mounted on slides. A Zeiss Observer Z1 fluorescence microscope was used to assess the percentage of phagocytized conidia. Because macrophage cells are not permeable, only internalized conidia remained unstained by CFW. At least 100 conidia were counted per sample, and a phagocytosis index was calculated.

For TNF-α measurements, macrophages (5 × 10^5^) were plated into 48-well plates for 16 hr at 37°, 5% CO_2_ in RPMI 140 media containing 10% FBS and 5% LCCM. For fungal infections, the strains were cultured for 18 hr up to a hyphal stage at a density of 2 × 10^4^ per well, and then they were UV-irradiated and used to stimulate the BMDMs. After infection, cells were centrifuged to synchronize the infection and allowed to stand and be infected for 18 hr. The supernatant was collected and the cytokine was measured by enzyme-linked immunosorbent assay (ELISA) with a mouse TNF-α kit (R&D Quantikine ELISA) according to the manufacturer’s instructions. For the positive control, 1 µg/ml of LPS from *E. coli* (Sigma) was used.

### Mouse model of invasive pulmonary aspergillosis and ethics statement

The virulence of *A. fumigatus* strains was analyzed using a murine model for invasive aspergillosis, as previously described (Dinamarco *et al.* 2012). For the negative control, a group of five mice received sterile PBS only. Mice were weighed every 24 hr starting from the day of infection and visually inspected twice daily. In the majority of cases, the endpoint for survival experimentation was identified when a 20% reduction in body weight was recorded, at which time the mice were killed. The statistical significance of comparative survival values was calculated by log rank analysis and the Prism statistical analysis package. This study and the protocols herein involving animal care were approved by the Local Ethics Committee for Animal Experiments from the Federal University of São Carlos - UFSCar (Permit Number: Protocolo CEEA n° 062/2009). All the animals were housed in groups of five within individually ventilated cages, and they were cared for in strict accordance with the principles outlined by the Brazilian College of Animal Experimentation [Sociedade Brasileira de Ciência em Animais de Laboratório - SBCAL (formerly COBEA - Colégio Brasileiro de Experimentação Animal)]. All efforts were made to minimize suffering. The animals were clinically monitored at least twice daily and humanely killed if moribund (as defined by lethargy, dyspnea, hypothermia, and weight loss). All stressed animals were killed by cervical dislocation.

### Data availability

Strains and plasmids are available upon request. Figure S1 contains detailed descriptions of all strains and constructions used in this study. The authors state that all data necessary for confirming the conclusions presented in the article are represented fully within the article.

## Results

### Identification of the RlmA homolog in A. fumigatus and construction of the ΔrlmA mutant and complemented strains

To identify the putative MADS box-family transcription factor gene that is involved in the maintenance of cell wall integrity in *A. fumigatus*, we searched the predicted ORF sequences of *A. fumigatus* strain Af293 by using BLASTp algorithms and the *A. nidulans* RlmA (AN2984) and *S. cerevisiae* Rlm1 protein sequences as queries. Searches with both RlmA and Rlm1 revealed a putative single ortholog in *A. fumigatus*, Afu3g08520 , which we subsequently designated RlmA to be consistent with previous nomenclature in *A. niger* and *A. nidulans* (Damveld *et al.* 2005; Fujioka *et al.* 2007). The RlmA protein sequence from *A. nidulans* and *A. fumigatus* shared 66.0% amino acid identity and 73.9% protein sequence similarity (e-value 0.0), while yeast Rlm1 and *A. fumigatus* RlmA shared a 62.4% amino acid identity and 71.8% (e-value 1e-27) protein sequence similarity. The *rlmA* gene model (available at http://www.aspgd.org) is supported by RT-PCR experiments that indicate a cDNA product of 1803 bp and a genomic sequence of 1929 bp (data not shown). The *rlmA* cDNA sequencing (A1163 strain) confirmed the existence of the two predicted introns as listed, one that was 74 bp in length and located between nucleotides 57 and 130 and one that was 52 bp long and located between nucleotides 288 to 339 (data not shown). In addition, we were able to detect a single nucleotide polymorphism in strain A1163 (AFUB_040580 ) in comparison with the Af293 strain at position 522 of the cDNA sequence, which results in a silent mutation from “CCC” (in the Af293 strain) to “CCG” (in the A1163 strain) that encodes a proline residue in both cases. The hypothetical protein that is encoded by *rlmA* is 600 amino acids long and has a calculated molecular weight of 64.4 kDa. The protein domain organization of *A. fumigatus* RlmA resembles that of yeast and other filamentous fungal RLM homologs (described in Damveld *et al.* 2005), indicating the presence of the highly conserved MADS box domain (amino acids 1–61) as assessed by Interpro analysis (http://www.ebi.ac.uk/interpro/; PROSITE entry PS50066 - MADS-box domain profile). These results strongly indicate that Afu3g08520 encodes the *A. fumigatus* MADS box transcription factor, RlmA.

To determine the role of RlmA in *A. fumigatus* cell wall homeostasis, we generated a deletion mutant by replacing the genomic sequence of *rlmA* with the *pyrG* selectable marker in the Δ*akuB*^KU80^ strain (Figure S1). The correct deletion of the *rlmA* gene in PCR-positive transformants was confirmed by Southern blot analysis (data not shown and Figure S1, A and B). To confirm that the phenotypes of the Δ*rlmA* strain were caused by loss of RlmA, we also generated a complemented strain through the ectopic reintroduction of the wild-type *rlmA* gene into the Δ*rlmA* mutant strain. The integration of the *rlmA* gene in the Δ*rlmA* mutant background was confirmed by PCR (Figure S1C). The complemented strain was indistinguishable in terms of growth and sporulation from the wild-type strain. Accordingly, the mRNA abundance of the *rlmA* gene was determined in the complemented strain by qRT-PCR and the *rlmA* expression was equivalent between the wild-type and Δ*rlmA*::*rlmA^+^* strains and absent in the Δ*rlmA* mutant (data not shown).

### RlmA is involved in the maintenance of cell wall integrity

We previously showed that the *A. fumigatus* PkcA is required for the activation of the CWI pathway via the MAP kinase MpkA (Rocha *et al.* 2015). In *S. cerevisiae*, the *RLM1* is the linking component between cell wall damage sensed by the PKC pathway and cell wall synthesizing enzymes. Therefore, we attempted to understand the role played by *rlmA* in maintaining cell wall integrity. We initially investigated the phenotypes related to cell wall and endoplasmic reticulum (ER) stress, and ion deprivation that could be observed upon *rlmA* loss-of-function.

**Figure 1:**
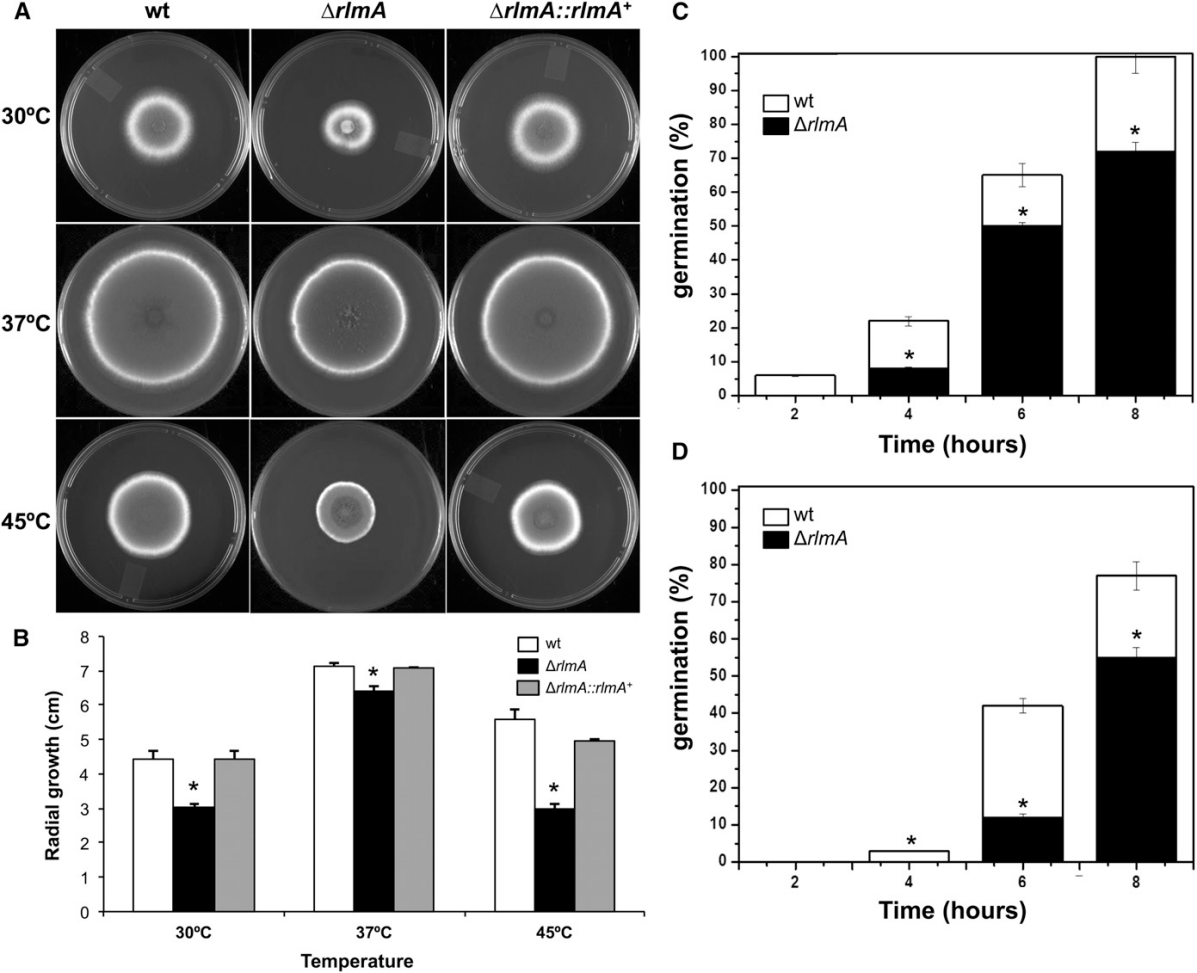
Growth phenotypes and germination rates of the Δ*rlmA* mutant. 1 × 10^4^ conidia of each strain were inoculated onto the center of complete solid medium, and their radial growth was measured after 72 hr at the indicated temperatures (A and B). 1 × 10^6^ conidia of each strain were inoculated in 2 ml of liquid YG culture and incubated at 37° (C) and 45° (D) for 2, 4, 6, and 8 hr before the percentage of germinated conidia were evaluated. The experiments were performed in triplicate, bars = SD, *P ≤ 0.01. SD, standard deviation; wt, wild-type.

The Δ*rlmA* strain exhibited decreased radial growth at 30°, 37°, and 45° on both complete (Figure 1, A and B) and minimal medium (data not shown). At 37°, the radial growth rate of the Δ*rlmA* strain was approximately 15% less than that of the wild-type and complemented strains, indicating that vegetative growth may be partially affected by the absence of RlmA. This finding is also reflected in the lower germination rates observed for the Δ*rlmA* strain at both 37° and 45°. When the germination was monitored, there was a significant decrease (*P* ≤ 0.01) in the emergence of germ-tubes in the Δ*rlmA* mutant by 30% and 25% at 37° and 45°, respectively, in comparison with that of the wild-type after 8 hr of growth (Figure 1, C and D).

**Figure 2:**
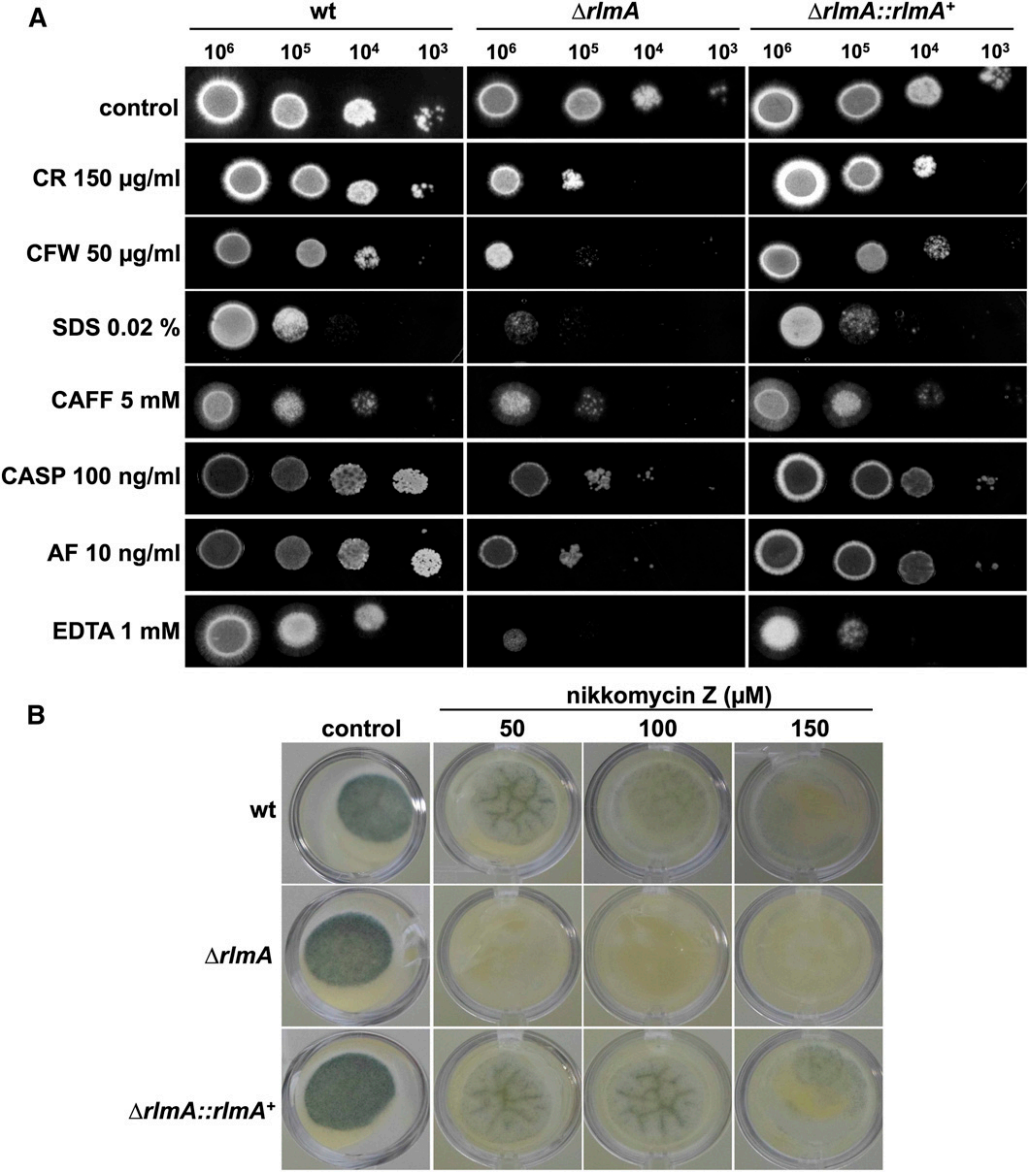
Sensitivity of Δ*rlmA* to cell wall-disturbing compounds. (A) The indicated number of conidia were inoculated onto solid YG plates that were supplemented with the following different cell wall perturbing agents: congo red (CR), calcofluor white (CFW), sodium dodecyl sulfate (SDS), caffeine (CAFF), caspofungin (CASP), anidulafungin (AF), and ethylenediaminetetraacetic acid (EDTA). (B) 1 × 10^4^ conidia/well were inoculated in YAG medium in a 24-well plate containing the indicated concentrations of nikkomycin Z. The plates were incubated for 48 hr at 37°. wt, wild-type.

Cell wall biosynthesis and/or remodeling was disturbed in the Δ*rlmA* strain because this mutant was more sensitive to the tested cell wall damaging compounds including CR, CFW, CAFF, CASP, AF, and nikkomycin Z, as well as SDS, which disrupts cell membranes (Figure 2, A and B). The mutant strain was also more sensitive to metal deprivation caused by the chelating agents EDTA (1 mM) and EGTA (300 mM) (Figure 2A and data not shown). Lower concentrations of CR and CFW were also tested, and they indicated that the decreased tolerance of the Δ*rlmA* strain to CFW and CR occurred when the concentrations were as low as 25 µg/ml and 30 µg/ml, respectively (Figure S3A). Increased sensitivity to CR, CFW, and CAFF could be rescued on minimal media containing the osmotic stabilizer D-sorbitol (1.2 M), which suggested a possible defect in the cell wall of the *rlmA* mutant (Figure S3B). We did not observe lysis phenotypes or conidia swelling at the microscopic level in the Δ*rlmA* mutant in the presence of cell wall damage caused by CR or CFW (data not shown). The CWI has been shown to be connected with ER stress and the Unfold Protein Response (UPR) during the biosynthesis or reinforcement of the cell wall (Richie *et al.* 2009; Malavazi *et al.* 2014; Rocha *et al.* 2015). Accordingly, the Δ*rlmA* strain was more sensitive to the ER-stressing agents BFA, DTT, and TM, and this phenotype on DTT could also be partially rescued by adding D-sorbitol (Figure S4). We also observed an increased susceptibility of the Δ*rlmA* strain to voriconazole and fluconazole (Figure S5). It is possible that the noticeable CWI defects in these mutants allow the potentialization of azoles efficiency, which is the premise for employing a combined therapy strategy with these drug classes.

**Figure 3:**
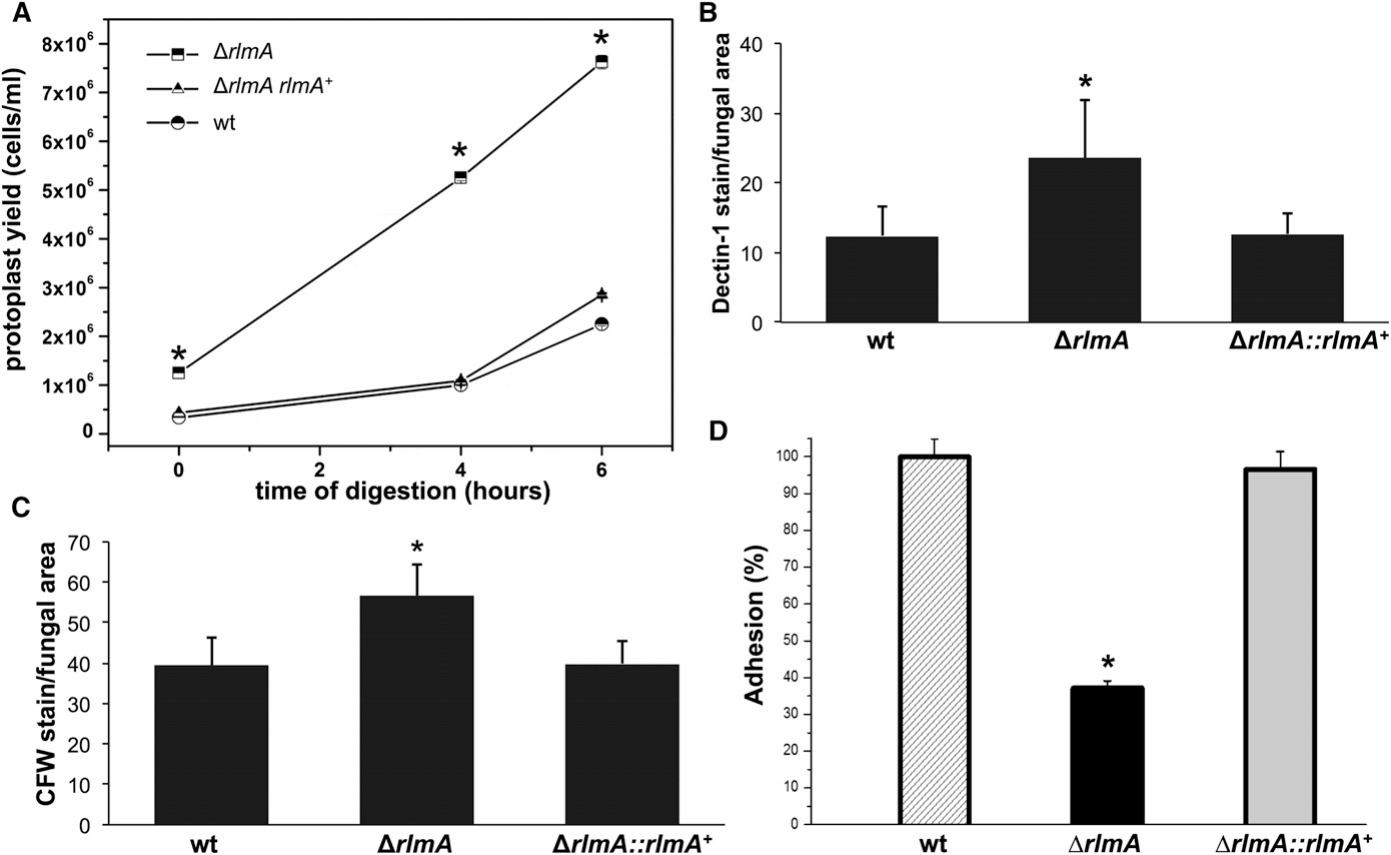
Loss of *rlmA* causes increased detection of β-1,3-glucan and chitin, enhanced cell wall enzymatic hydrolysis, and decreased biofilm formation. (A) A 100 mg quantity of mycelium was incubated in 50 ml of the digestion mixture at the indicated incubation times. Protoplasts were quantified in a Neubauer chamber (n = 4; bars = SD, *P ≤ 0.01). Conidia were cultured in liquid medium to the hyphal stage, fixed, and stained with soluble dectin-1 or CFW to detect the content of exposed β-glucan (B) or chitin (C), respectively. The staining intensity was calculated by averaging the amount of staining relative to the total area of each fungal cell (n = 100) using ImageJ software. The experiments were performed in triplicate. Bars = SD, *P ≤ 0.05. (D) Biofilm formation was evaluated by crystal violet absorbance at 570 nm and expressed as the percentage of adhesion considering 100% for the wild-type strain. The experiments were performed in quintuplicate. Bars = SD, *P ≤ 0.05. CFW, calcofluor white; SD, standard deviation; wt, wild-type.

**Figure 4:**
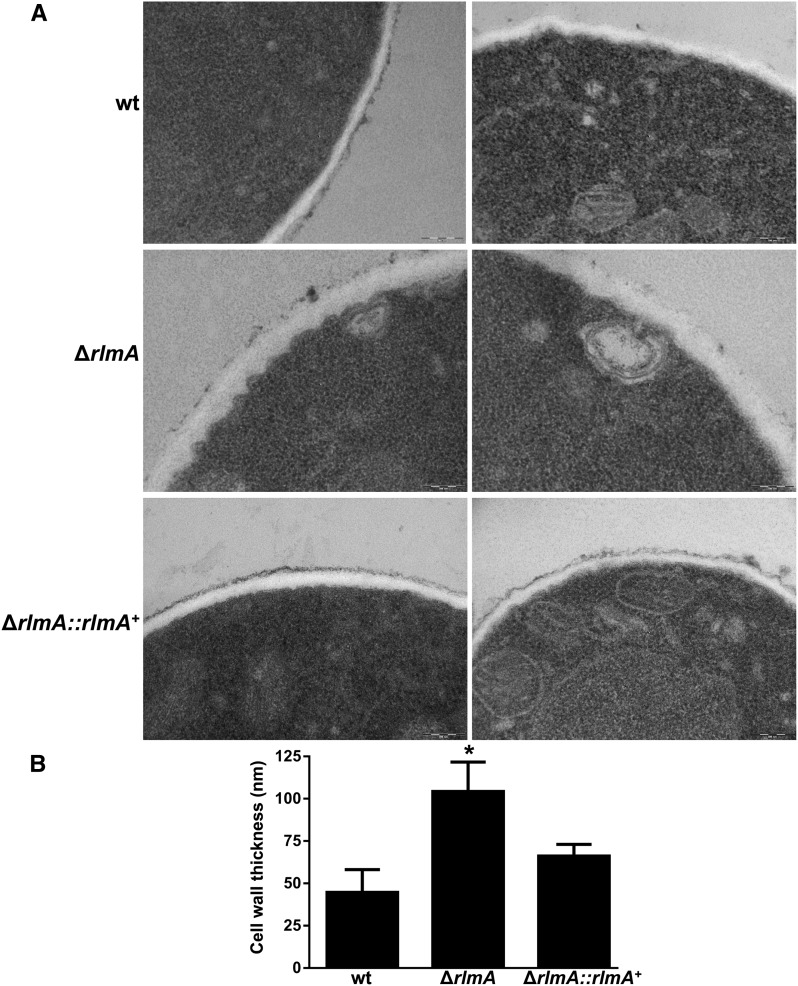
Δ*rlmA* germlings present thicker cell walls. (A) The wild-type, Δ*rlmA*, and Δ*rlmA*::Δ*rlmA^+^* strains were grown in complete medium and prepared for TEM analysis. Bars = 200 nm. (B) Cell wall thickness measurements of the germlings. Values are expressed as mean ± SD of 50 sections of different germlings. *P ≤ 0.05 (One Way ANOVA). ANOVA, analysis of variance; SD, standard deviation; TEM, transmission electron microscope; wt, wild-type.

As an indirect approach to investigate the cell wall composition and architecture of the Δ*rlmA* strain, the hyphae of the Δ*rlmA*, wild-type, and complemented strains were subjected to enzymatic digestion. The protoplasts were then harvested and counted. The digestion of the Δ*rlmA* mutant yielded approximately four times more protoplasts than the wild-type and complemented strains (Figure 3A) after 4 and 4 hr of digestion, indicating that the Δ*rlmA* mutant cell wall was much more susceptible to enzymatic degradation. To investigate if this carbohydrate modification affected the cell wall organization, the β-1,3-glucan and chitin levels were assessed in these three strains via soluble dectin-1 and CFW staining, respectively. The intensity of the dectin-1 and CFW staining per fungal area was 91.8% and 43.5% higher in the Δ*rlmA* mutant, respectively, than it was in the wild-type and complemented strains (Figure 3, B and C). Because the altered composition of the cell wall can impact the adhesion properties of the fungal cell, especially upon interaction with the host tissues, we subsequently investigated the ability of the Δ*rlmA* mutant to promote adhesion to abiotic surfaces by measuring adhesion of mature hyphae in polystyrene plates. The adhesion was significantly reduced (65%) in the mutant when compared with the wild-type and complemented strains (Figure 3D). We also investigated the cell wall organization in the Δ*rlmA* mutant by inspecting the thickness of the cell wall in this strain by transmission electron microscopy (TEM). Interestingly, the Δ*rlmA* germlings were about twofold thicker than the wild-type and the complementing strains (Figure 4).

Taken together, these results suggest that the mutant strain possesses a modified cell wall organization that affects several responses to the environmental stimuli.

### The CWI pathway genes pkcA, mpkA, and rlmA interact genetically in A. fumigatus

**Figure 5:**
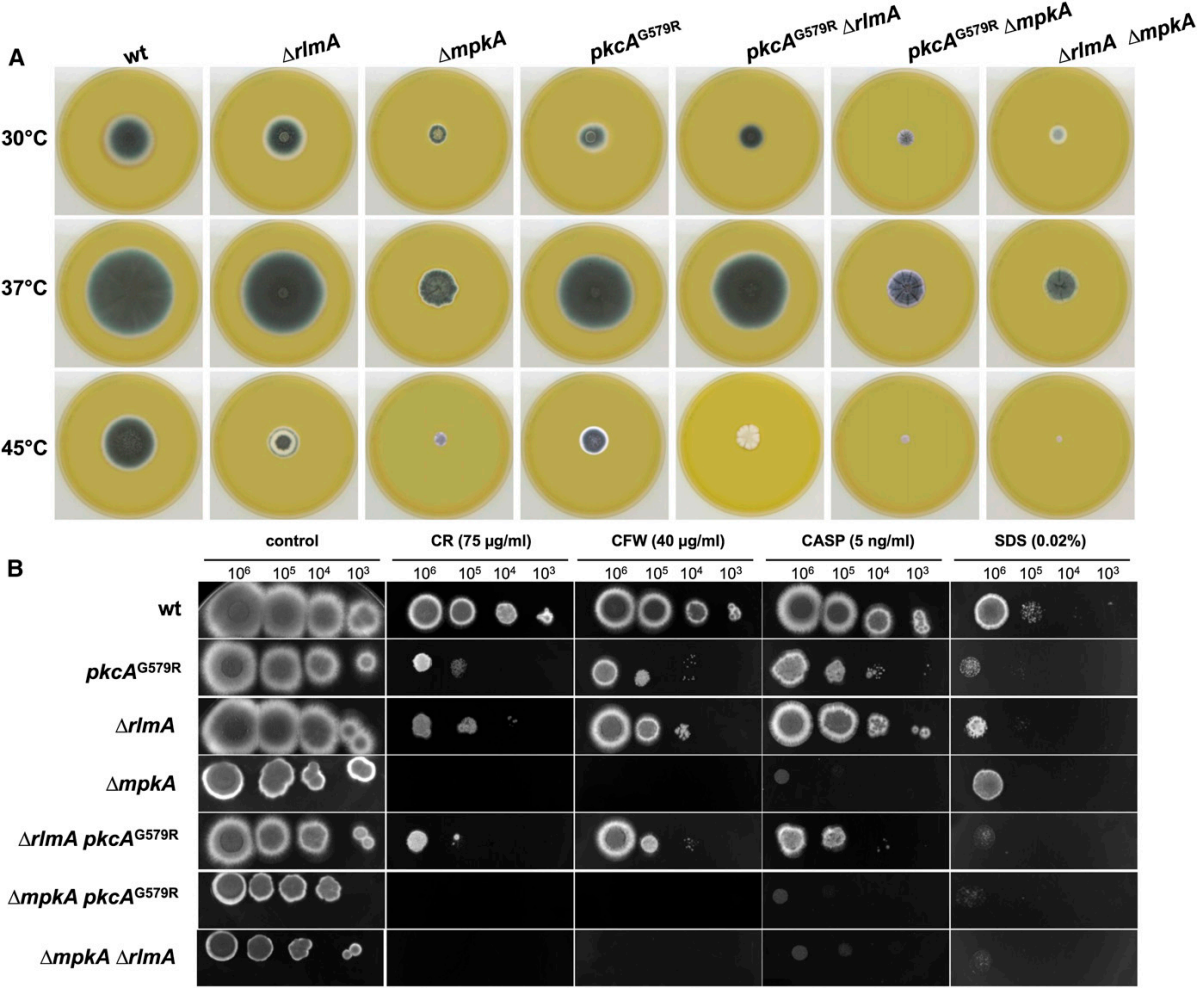
*rlmA*, *pkcA*, and *mpkA* interact genetically during cell wall stress. (A) The growth phenotypes and colony morphology of the CWI pathway single and double mutants. 1 × 10^5^ conidia were inoculated onto the center of the YAG medium and incubated at different temperatures for 72 hr. (B) The indicated numbers of conidia were spotted onto solid YG plates that were supplemented with congo red (CR), calcofluor white (CFW), caspofungin (CASP), and sodium dodecyl sulfate (SDS). The plates were incubated for 72 hr at 37°. Genetic interactions were determined by evaluating the sensitivity to CR, CFW, CASP, or SDS in the double deficient mutants relative to that of the parental single mutants. CWI, cell wall integrity; wt, wild-type.

We previously showed that MpkA acts downstream of PkcA (Rocha *et al.* 2015). However, it is still not established that RlmA is the MADS Box transcription factor that is ultimately activated by phosphorylated MpkA following cell wall damage in *A. fumigatus*. As a preliminary step to address this question, we investigated the possible genetic interactions among *pkcA*, *mpkA*, and *rlmA* during cell wall damage. Accordingly, we constructed the double mutants Δ*rlmA pkcA*^G579R^ (*pkcA*^G579R^ is a mutant strain where *pkcA* has attenuated activity, Rocha *et al.* 2015), Δ*mpkA* Δ*rlmA*, and Δ*mpkA pkcA*^G579R^ (Figure S1, D–F). The vegetative growth and colony morphologies of the double mutants on YG medium are shown in Figure 5A. Interestingly, the three double mutants cannot conidiate at 45°. The epistasis grouping of these mutants was determined by evaluating the sensitivity of the double deficient mutants to CR, CFW, CASP, or SDS in comparison with that of the parental single mutants. The double mutant Δ*rlmA pkcA*^G579R^ was inhibited to the same extent as the corresponding parental strains in the presence of CR, CFW, CASP, and SDS, indicating that *pkcA* and *rlmA* display the expected epistatic interaction (Figure 5B and Figure S6, A–D). The double mutant Δ*mpkA* Δ*rlmA* also seems to be genetically interacting in the presence of CR and CFW, but not in the presence of SDS, because the Δ*mpkA* strain is intrinsically more resistant to SDS. The phenotype of the double mutant *pkcA*^G579R^ Δ*mpkA* resembles that of the Δ*mpkA* strain. The data show that, in the presence of CR, CFW, and CASP (cell-wall agents that perturb polymer bonding or β-1,3 glucan synthesis), deletion of *mpkA* has a dominant phenotype over the *pkcA*^G579R^, Δ*rlmA*, or *pkcA*^G579R^ Δ*rlmA* mutants. In SDS (a membrane-perturbing agent but also can denature cell wall proteins), it is *rlmA* deletion that has the dominant phenotype over the Δ*mpkA*, *pkcA*^G579R^, and *pkcA*^G579R^ Δ*mpkA* mutants. Taken together, these results suggest that resistance to cell wall perturbing agents requires MpkA and that the pathway that is important for SDS resistance requires RlmA. Thus, the SDS resistance pathway could feed into the CWI pathway below MpkA, at RlmA.

### RlmA positively regulates the phosphorylation of MpkA and is induced both at the protein and transcriptional levels during cell wall stress

**Figure 6:**
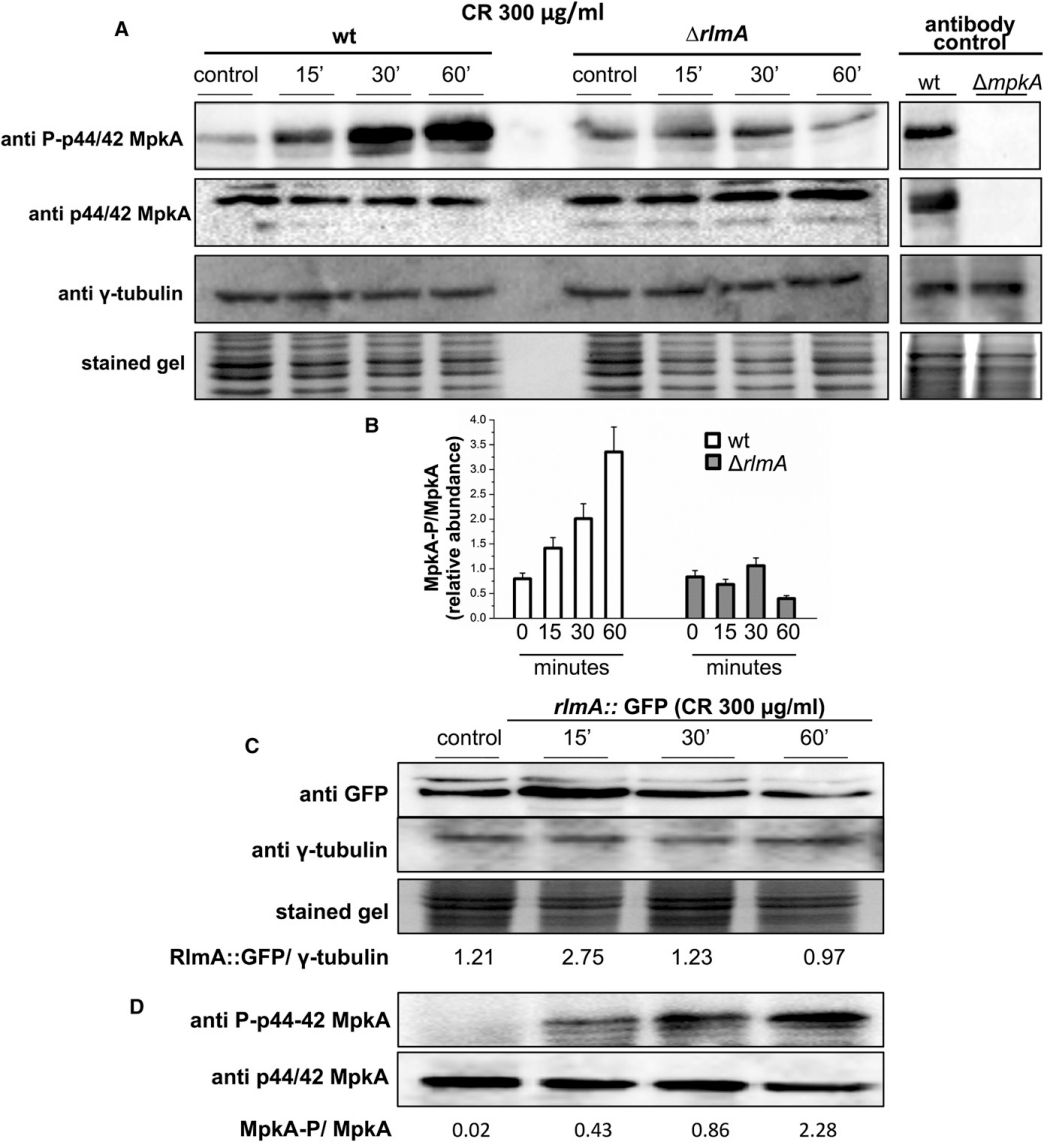
The Δ*rlmA* mutant strain has impaired CWI pathway activation. (A) Conidia from the wild-type and mutant strain were grown for 16 hr in liquid medium and cell wall stress was induced through exposure to CR for 0, 15, 30, and 60 min. The phosphorylated fractions and the total MpkA amount were detected using anti-phospho p44/42 MAPK and anti-p44/42 MAPK antibodies, respectively. No detection is observed in the Δ*mpkA* mutant (antibody control). The γ-tubulin antibody and the Coomassie Brilliant Blue stained gel were used as loading sample controls (A). Densitometry analysis of western blots showing the ratio of phosphorylated MpkA/nonphosphorylated MpkA in the wild-type and Δ*rlmA* mutant strain expressed as the relative abundance (B). The conidia of the *rlmA*::*gfp* strain were cultured under the same conditions as those described in (A). Protein extracts from different time points were subjected to western blots to detect the total amount of RlmA using anti-GFP antibodies. Signal intensities were quantified as the RlmA::GFP/γ-tubulin pixel intensity ratio (C). The *rlmA*::*gfp* strain was cultured under the same conditions as those described in (C) and subjected to western blot analysis to detect both the phosphorylated fraction and the total MpkA protein as described in (A). CR, congo red; CWI, cell wall integrity; GFP, green fluorescent protein; MAPK, mitogen-activated protein kinase; wt, wild-type.

The CR-induced phosphorylation of MpkA has been shown to be dependent on PkcA because either the loss-of-function or the pharmacological inhibition of PkcA leads to lower levels of MpkA phosphorylation (Bom *et al.* 2015; Rocha *et al.* 2015). We also show that *pkcA*, *mpkA*, and *rlmA* are genetically interacting during the cell wall stress caused by CR (Figure 5). Therefore, we sought to investigate the MpkA phosphorylation effect under these conditions in the Δ*rlmA* mutant strain. The phosphorylation level of the MpkA protein was determined using the antiphospho-p44/42 and p44/42 MAPK antibodies directed against phosphorylated and total MpkA. These antibodies recognize a single band in the wild-type and no band in the Δ*mpkA* mutant. As expected, the MpkA protein was phosphorylated in response to CR presenting 1.5-, 2.0-, and 3.5-fold increases after 15, 30, and 60 min, respectively (Figure 6). Surprisingly, the phosphorylation of MpkA did not increase over time in the Δ*rlmA* mutant post-CR treatment. These results suggest that there is a positive regulatory role for *rlmA* during the cell wall stress that could intensify the activity of the CWI pathway, and that the ultimate effect is increased MpkA phosphorylation. To assess RlmA protein abundance during cell wall stress, we generated an RlmA::GFP strain that employs its endogenous promoter by replacing the wild-type *rlmA* allele. This resulting strain behaves exactly like the wild-type (data not shown). In addition, as an additional control procedure, the transformation of the *rlmA*::*gfp* cassette into the Δ*rlmA pyrG*^-^ mutant strain yielded complemented strains that were able to fully rescue the cell wall phenotypes of the Δ*rlmA* mutant (data not shown). To verify that the total RlmA amount is modulated in the presence of cell wall stress, western blot analyses were performed using a commercially available anti-GFP antibody. Protein extracts from RlmA::GFP that were cultivated under cell wall stress conditions induced by CR were analyzed. There was an increase of approximately 2.4-fold in the RlmA protein expression after 15 min of CR exposure (Figure 6C). The functional RlmA::GFP strain consistently presents wild-type levels of MpkA phosphorylation under the same conditions (Figure 6D). Although, we were able to identify the RlmA::GFP by western blot analysis, the GFP signal was not strong enough to allow us to verify the subcellular localization of RlmA::GFP (data not shown).

**Figure 7:**
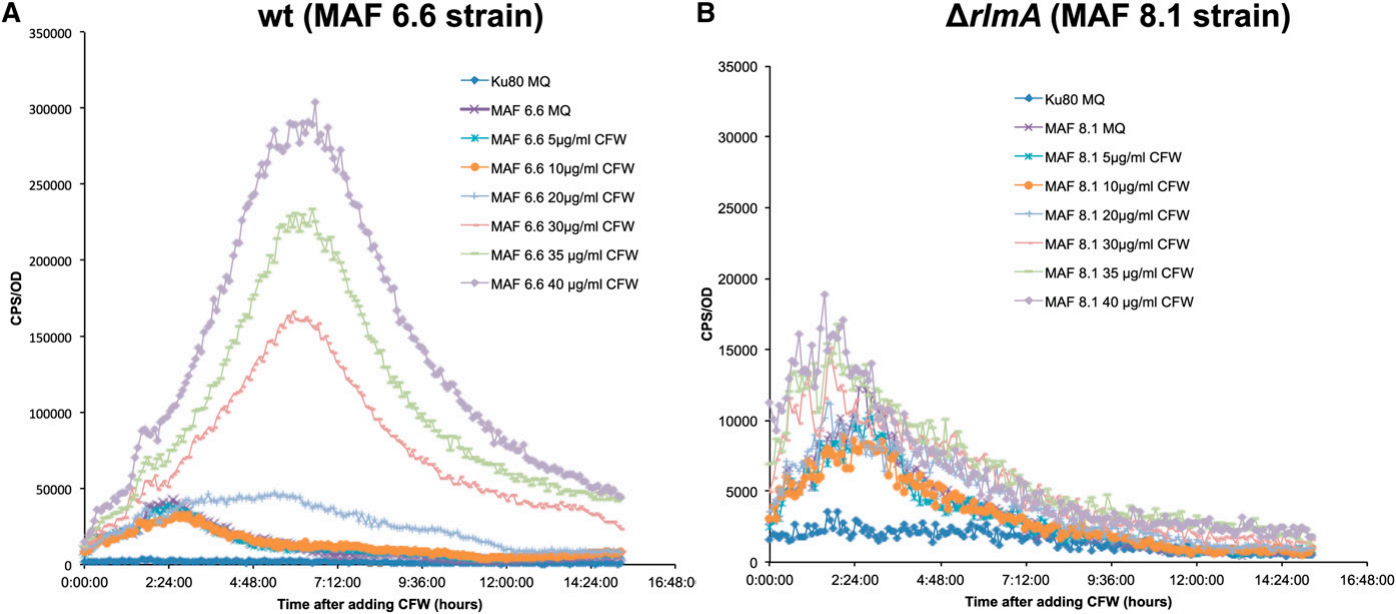
Lux activity assay of *A. fumigatus* MAF 6.6 (wt; P*agsA*::*mluc*) and MAF 8.1 (Δ*rlmA*; P*agsA*::*mluc*) incubated with different concentrations of CFW. (A) CPS (luciferase activity) per OD_600_ measured during incubation of *A. fumigatus* control strain (Δ*akuB*^KU80^ without lux reporter) with MQ (deionized water) and MAF 6.6 in the presence of 5-40 μg/ml of CFW after 15 hr of incubation. (B) CPS (luciferase activity) per OD_600_ measured during incubation of *A. fumigatus* control strain (Δ*akuB*^KU80^ without lux reporter) with MQ (deionized water) and MAF 8.1 (Δ*rlmA*; P*agsA*::*mluc*) in the presence of 5–40 μg/ml CFW after 15 hr of incubation. The results were normalized by the number of conidia (1.5 × 10^4^ per assay) and are expressed as at least three repetitions. Calcofluor white, CFW; CPS, counts per second; OD, optical density; wt, wild-type.

As an additional approach to understand the function of *A. fumigatus rlmA* in activating genes involved in cell wall maintenance, we constructed a strain containing the luciferase (*mluc*) reporter gene under the control of the *A. niger agsA* (α-1,3-glucan synthase) promoter to monitor and measure temporal changes in the *agsA* expression. This approach was chosen because in *A. niger*, RlmA, and the RlmA box domain [TA(A/T)_4_TAG] in the *agsA* promoter are required to induce the *agsA* gene in the presence of CFW (Damveld *et al.* 2005). Accordingly, two strains were generated, one in which the *pyrG* locus of the wild-type (*akuB*^KU80^) strain was replaced by the *PagsA*::*mluc* cassette (MAF 6.6) and another where the *pyrG* locus of the Δ*rlmA* strain was replaced by the *PagsA*::*mluc* cassette (MAF 8.1). The luciferase activity of these strains was determined in a luciferase activity assay in the presence of CFW-induced cell wall stress (Figure 7). The results show that the luciferase activity was ∼5–20-times higher in the MAF 6.6 strain in a CFW dose-dependent manner compared to the MAF 8.1 strain. These results indicated that *A. fumigatus* RlmA can bind to the RlmA binding motif and activate the expression of luciferase upon cell wall damage, which further suggests a direct role for *rlmA* in governing the expression of genes involved in the cell wall stress response.

Subsequently, the wild-type, Δ*rlmA*, and Δ*mpkA* strains were used to assess the mRNA steady-state levels of the *rlmA* gene and the other cell wall-related genes under CR stress conditions. This approach aimed to understand the transcriptional alterations that eventually occur in the cell when the function of either the *rlmA* transcription factor or the MAP kinase *mpkA* is absent, which could explain the increased sensitivity of the Δ*rlmA* and Δ*mpkA* mutants to cell wall stressing agents. Accordingly, we examined the mRNA levels of *pkcA*, *mpkA*, and the primary genes that encode cell wall biosynthesis enzymes as follows: the catalytic subunit of the β-1,3-glucan synthase (*fksA*); α-1,3-glucan synthases (*agsA-C*); 1,3-β-glucanosyl transferases (*gelA-C* and *gel4*); and the eight *A. fumigatus* chitin synthases (*chsA-G* and *csmB*).

**Figure 8:**
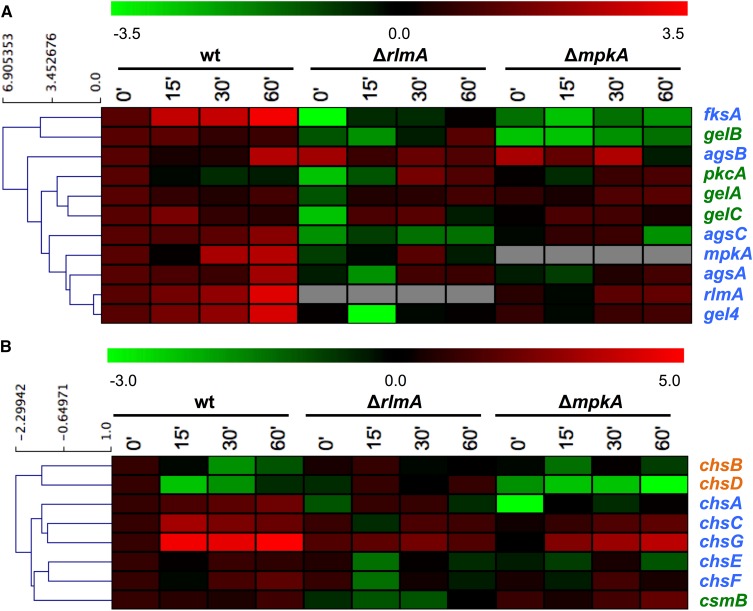
Hierarchical clustering analysis showing the gene expression profiles of wild-type, Δ*rlmA*, and Δ*mpkA* mutant strains to CR-induced cell wall stress. The relative levels of each transcript were monitored by qRT-PCR of mRNA extracted after 0, 15, 30, and 60 min of CR exposure. The values were log_2_ transformed and submitted to a hierarchical clustering algorithm (Euclidian distance) by using the TMEV software (available at http://www.tm4.org/mev.html). The color scheme was used to designate genes that were down-regulated (green) or up-regulated (red). Color of the gene names indicates the wild-type response to CR-induced cell wall stress, *i.e.*, responders (blue); poor/limited responders (green); and negative responders (orange). (A) Miscellaneous genes. (B) Chitin synthase genes. Figure S7 shows the bar graphs and the relative expression value of each time point. CR, congo red; mRNA, messenger RNA; qRT-PCR, quantitative real-time polymerase chain reaction; wt, wild-type.

**Figure 9:**
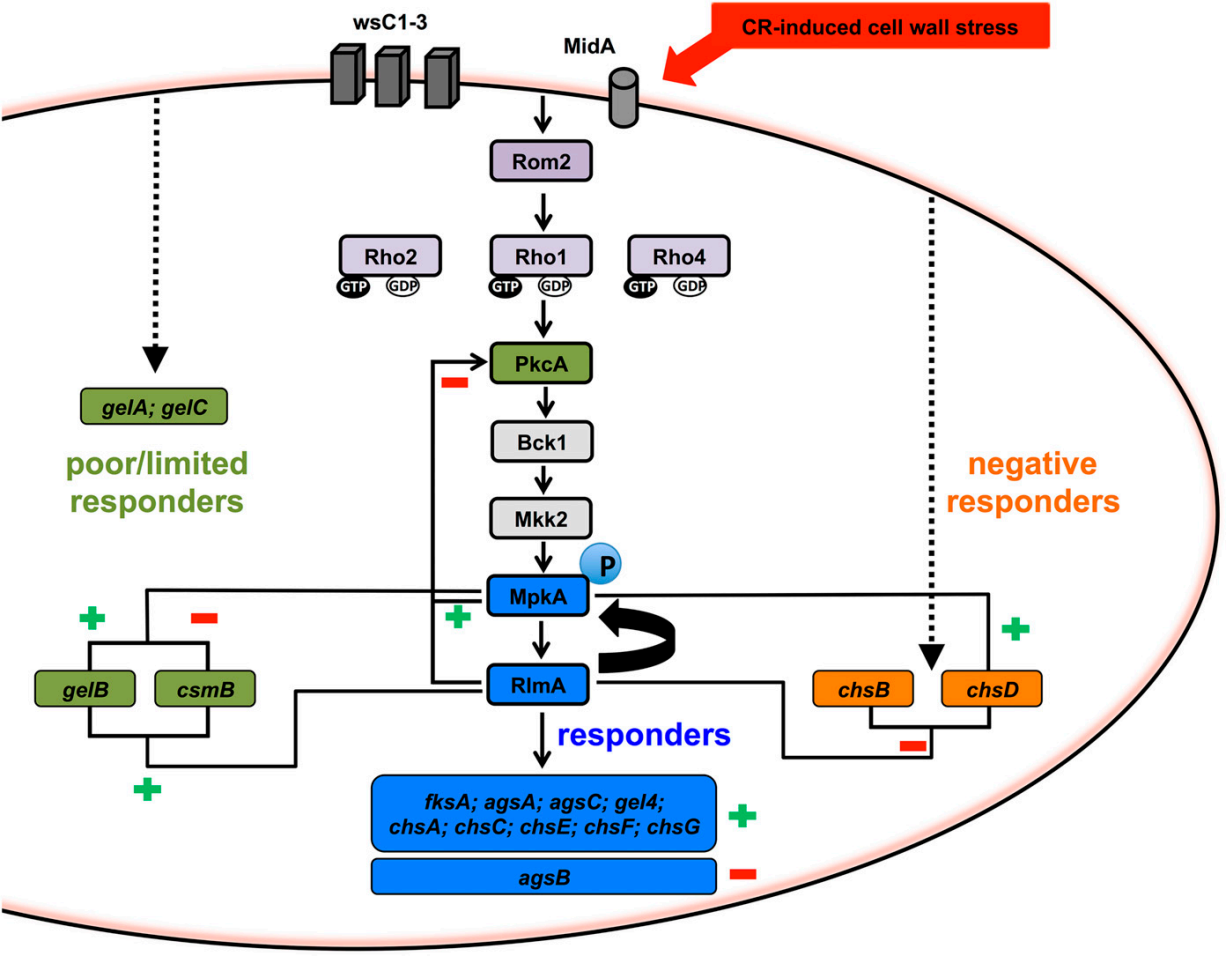
Diagram indicating the proposed regulation for activation/inhibition or nonconnection between *rlmA* and *mpkA* and the cell wall biosynthesis genes during cell wall stress induced by CR. *midA* is the mechanosensor primarily involved in sensing cell wall stress induced chitin-binding agents such as CR. The connection with the CWI pathway PkcA/Bck1-Mkk2-MpkA epistatic module is mediated by the guanine nucleotide exchange factor *rom2* and the Rho GTPase *rho1*, which are involved in MpkA phosphorylation. The blue boxes indicate genes that respond to CR and are thus transcriptionally regulated. The green boxes indicate genes grouped in the category of poor/nonresponders to CR that results in limited or no transcriptional responses to CR. The orange boxes represent the negative responder genes (down-regulated) to CR. The symbols (+) and (−) shown in green or red indicate a regulatory consequence of *rlmA* and/or *mpkA* in positively or negatively regulating (respectively) transcription of target genes. Curved arrow indicates the positive feedback regulatory effect of *rlmA* in maintaining the MpkA phosphorylation during the CR-induced cell wall stress. Dashed lines indicate additional hypothetical signaling transduction mechanism. This diagram is based on data from this article (Figure 5, Figure 6, Figure 7, and Figure 8), Dichtl *et al.* (2012), Samantaray *et al.* (2013), Rocha *et al.* (2015) and Dichtl *et al.* (2016). CR, congo red; CWI, cell wall integrity.

Overall, in wild-type cells, the response to CR can be divided in three categories: (i) the “responders,” which are genes up-regulated by CR including *rlmA*, *mpkA*, *fksA*, *agsA-B-C*, *gel4*, and *chsA-C-E-F-G*; (ii) the “poor or nonresponder” genes, which show limited or no response to CR including *pkcA*, *gelA-B-C*, and *csmB*; and (iii) the “negative responders,” which are genes down-regulated by CR including *chsB* and *chsD* (Figure 8, A and B and Figure S7, A and B). The results show that there are much more variable patterns for the expression of the cell wall-related genes in the genetic background of the Δ*rlmA* and Δ*mpkA* mutant strains. Hence, we grouped the genes aiming to show the activation/inhibition or nonconnection between *rlmA* and *mpkA* and the cell wall biosynthesis genes, and summarized the findings in the diagram displayed in Figure 9. The responder genes (blue letters and blue boxes in Figure 8 and Figure 9, respectively) are all transcriptionally regulated by both RlmA and MpkA, all positively except for *agsB*, the expression of which was significantly enhanced in both the Δ*rlmA* and Δ*mpkA* strains, especially in the absence of CR (3.7- and 3.9-fold increase, respectively, Figure S7A). Of the poor responders to CR (shown in the green letters and green boxes in Figure 8 and Figure 9, respectively), *pkcA* is negatively regulated by both *rlmA* and *mpkA*, while the chitin synthase *csmB* is positively regulated by *rlmA* and negatively regulated by *mpkA*. In addition, *gelB* is positively regulated by both *rlmA* and *mpkA*. Interestingly, *gelA* and *gelC* seem not to be controlled by the MpkA-RlmA signaling circuit during exposure to CR. Concerning the two negative responder genes (*chsB* and *chsD*; showed in orange letters and orange boxes in Figure 8 and Figure 9, respectively), both are also negatively regulated by *rlmA* while *chsD* is positively regulated by *mpkA* only (see also Figure S7B for expression values).

Regarding the regulator genes *mpkA* and *rlmA* (responders to CR: blue letters and blue boxes in Figure 8 and Figure 9, respectively), the transcriptional activation of *rlmA* in the wild-type strain is accompanied by an increase in the RlmA protein levels (see Figure 6C). By contrast, this increase in *rlmA* mRNA abundance is significantly reduced in the Δ*mpkA* strain in comparison with the wild-type (2.2-fold after 60 min of CR treatment, Figure S7A), suggesting that the activation of *rlmA* in response to CR depends on PkcA-MpkA signaling. Likewise, there is also lower abundance of *mpkA* transcripts in the Δ*rlmA* mutant in comparison with the wild-type strain, correlating with the lower levels of MpkA phosphorylation observed in Figure 6A.

Collectively, these results suggest that there is a positive feedback loop between MpkA and RlmA, and that these genes are involved in the transcriptional activation of several cell wall-related genes and also in the regulation of the CWI pathway.

### RlmA plays a role in the tolerance to oxidative stress

**Figure 10:**
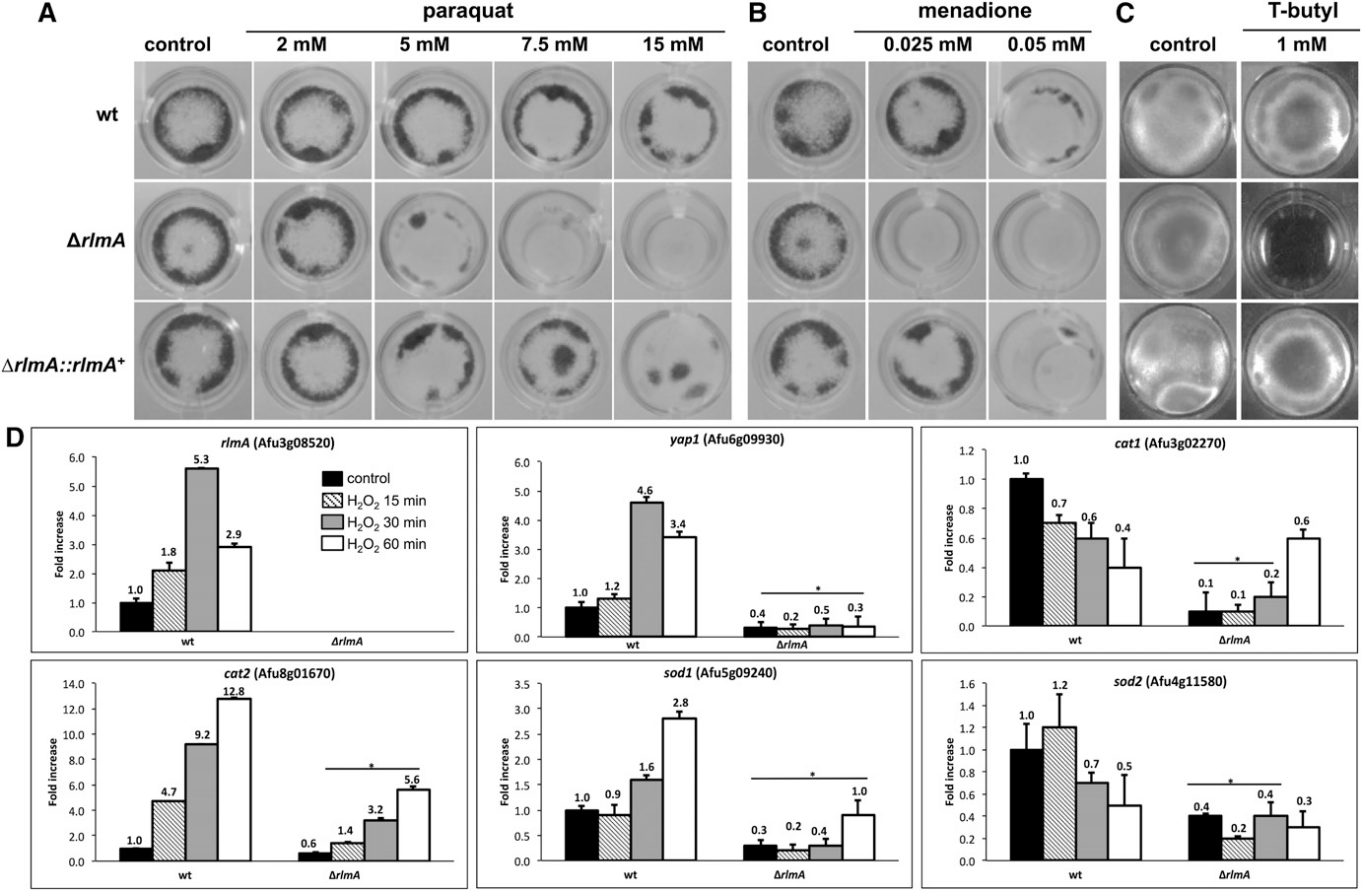
The Δ*rlmA* strain has increased susceptibility to oxidative stressing agents. 1 × 10^5^ conidia of each strain were inoculated into 24-well plates containing 1 ml of liquid MM and varying concentrations of paraquat (A), menadione (B), or T-butyl hydroperoxide (C) for 48 hr at 37°. (D) Quantitative real-time RT-PCR analysis of *A. fumigatus rlmA*, *yapA*, *cat1*, *cat2*, *sod1*, and *sod2*. The strains were grown for 24 hr in liquid MM medium. After incubation, H_2_O_2_ was added and the cultures and grown for an additional 15, 30, and 60 min. The mRNA abundance for each gene was normalized to β-tubulin in each strain, and the normalized mRNA abundance levels are relative to the wild-type strain at time point 0 (*i.e.*, prior to H_2_O_2_ treatment). The data represent the average value of two biological replicates, each of which was repeated in duplicate in the same run. Bar = SD, * *P* ≤ 0.05 (One Way ANOVA). ANOVA, analysis of variance; H_2_O_2_, hydrogen peroxide; mRNA, messenger RNA; RT-PCR, reverse transcription polymerase chain reaction; SD, standard deviation; wt, wild-type.

It has been demonstrated that CWI genes belonging to the circuit PKC-MAPK in *Aspergillus* species are important to sustain the oxidative stress induced by free radicals originating mainly from hydrogen peroxide, menadione, and paraquat (Valiante *et al.* 2008, 2009; Dirr *et al.* 2010; Kovacs *et al.* 2013). Accordingly, we investigated the mutant responses to oxidative damage to obtain information about whether *rlmA* could be indirectly involved in Reactive Oxygen Species (ROS) tolerance. The *rlmA* mutant showed increased sensitivity to paraquat, menadione, and *t*-butyl hydroperoxide in liquid MM (Figure 10, A–C). In addition, the Δ*rlmA* mutant was also more sensitive to hydrogen peroxide when the strains were evaluated in a zone inhibition assay in the presence of H_2_O_2_ 8% (*P* < 0.01). The inhibition zone diameters that were obtained for each strain were 10 ± 0.1 mm, 17 ± 0.1 mm, and 9 ± 0.1 mm for the wild-type, mutant, and complemented strain, respectively. We subsequently used a qRT-PCR approach to investigate the mRNA accumulation of *rlmA* as well as that of several genes known to be involved in the oxidative response in *A. fumigatus*, in the presence of H_2_O_2_ (5 mM). These genes include (i) *yapA*, which encodes the transcription factor homolog of yeast *YAP1* that is required for oxidative damage resistance (Lessing *et al.* 2007; Qiao *et al.* 2008); (ii) *cat1*, which encodes a mycelial catalase; (iii) *cat2*, which encodes a mycelial bifunctional catalase-peroxidase (Paris *et al.* 2003; Lessing *et al.* 2007; Sugui *et al.* 2008); (v) *sod1*, which encodes a Cu/Zn superoxide dismutase; and (v) *sod2* which encodes a manganese-superoxide dismutase (Holdom *et al.* 2000; Lessing *et al.* 2007; Sugui *et al.* 2008; Lambou *et al.* 2010). Interestingly, *rlmA* was up-regulated during H_2_O_2_ exposure (by 1.8, 5.3, and 2.9 times after 15, 30, and 60 min, respectively) (Figure 10D). Overall, *yapA*, *cat2*, and *sod1* showed increased mRNA abundance in the wild-type, whereas they were decreased in the Δ*rlmA* mutant suggesting that RlmA is a positive regulator of these genes. Taken together, these results indicate that RlmA contributes to the transcriptional regulation of several genes related to *A. fumigatus* oxidative stress adaptation.

### The ΔrlmA mutant is avirulent in a low-dose neutropenic murine infection model and leads to increased TNF-α levels and macrophage recognition

**Figure 11:**
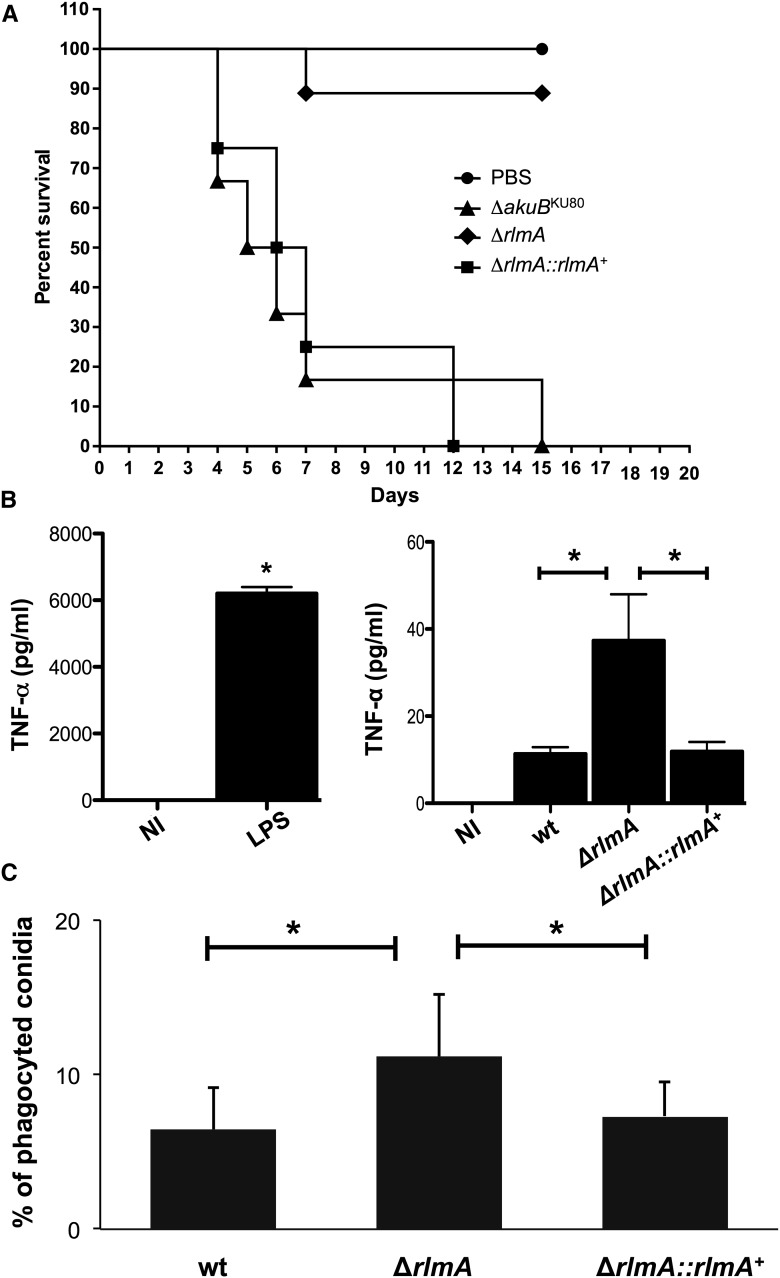
*A. fumigatus* RlmA is required for full virulence in a murine model of invasive pulmonary aspergillosis and for the activation of innate immunity against *A. fumigatus*. (A) BALB/c mice were immunocompromized with cyclophosphamide and hydrocortisone acetate and then inoculated with 2.5 × 10^6^ conidia intranasally. Compared to wild-type and the Δ*rlmA*::*rlmA*^+^ complemented strain, the Δ*rlmA* mutant showed a significant attenuation in virulence as measured by murine survival (*P* < 0.0001 by log-rank analysis). The survival difference between the wild-type and Δ*rlmA*::*rlmA*^+^ was not statistically significant (*P* > 0.1 by log-rank analyses). (B) The secretion of TNF-α from BMDM after coincubation with the *A. fumigatus* hyphae of wild-type, Δ*rlmA*, and Δ*rlmA*::*rlmA*^+^ complemented strains. The TNF-α levels were quantified by ELISA from the culture supernatant after 18 hr of BMDM infection. The data show the average ± SD (*P ≤ 0.005, One Way ANOVA). NI, noninfected; LPS, lipopolysaccharide (positive control). (C) The phagocytosis index is increased in the Δ*rlmA* mutant strain in comparison with the wild-type and Δ*rlmA*::*rlmA*^+^ complemented strain (average ± SD *P ≤ 0.05). Bone marrow-derived macrophages, BMDM; ELISA, enzyme-linked immunosorbent assay; PBS, phosphate-buffered saline; SD, standard deviation; wt, wild-type.

Subsequently, we evaluated the virulence and pathogenicity of the wild-type, Δ*rlmA*, and complemented strains in a low-dose murine infection model. The wild-type infection resulted in 100% mortality at 15 d postinfection, and the Δ*rlmA* infection caused a significantly reduced mortality rate of 10% at 15 d postinfection (Figure 11A, *P* ≤ 0.0001 and *P* ≤ 0.0001 for the comparison between the wild-type and the deletion mutant; Log-rank, Mantel–Cox, and Gehan–Breslow–Wilcoxon tests, respectively). Virulence was restored in an independent strain that resulted from a single ectopic reintegration of the wild-type *rlmA* sequence, and there were no significant differences between the wild-type and the complemented Δ*rlmA*::*rlmA^+^* strain.

The *rlmA* null mutant has an impaired response to cell wall stress. Thus, we reasoned that these phenotypes could impact the host immune response. Accordingly, we used Bone Marrow-Derived Macrophages (BMDMs) to measure the levels of proinflammatory cytokine Tumor Necrosis Factor α (TNF-α) that were released by these cells after coincubation with *A. fumigatus* conidia. TNF-α is an important inflammatory mediator that is secreted by macrophages when exposed to *A. fumigatus* or other fungal pathogens (Taramelli *et al.* 1996; Mehrad *et al.* 1999). BMDMs that were cocultured with the Δ*rlmA* strain showed approximately 3.5-fold higher TNF-α production than the wild-type or the complemented strain (*P* ≤ 0.005; Figure 11B). We also tested the ability of BMDMs to internalize wild-type, mutant, and complemented strain conidia. Approximately 11% of the Δ*rlmA* strain conidia were phagocytized after 80 min of coincubation. By contrast, only 6.4% of the wild-type and complemented strain conidia were internalized (*P ≤ 0.05; Figure 11C). These results suggest that the effect caused by the *rlmA* loss-of-function on the *A. fumigatus* CWI is important for macrophage recognition and inflammatory responses. Taken together, these results indicate the preeminent effect of RlmA on the virulence and pathogenicity of *A. fumigatus*.

## Discussion

The maintenance of cell wall integrity is one of the fundamental events that allow fungal cells to cope with different sorts of stresses that can perturb cell wall organization, ultimately preventing cell lysis and death. Cell wall biosynthesis in fungi is controlled by the CWI pathway, which is a signaling transduction cascade that amplifies the upcoming signal and mediates the downstream cell metabolic responses (Levin 2011). The CWI pathway is precisely regulated, and in *S. cerevisiae* it starts with the activation of the Pkc1-Mpk1 circuit and culminates with the activation of the Rlm1 transcription factor (Watanabe *et al.* 1997). This event causes the transcriptional activation of several cell wall biosynthesis genes (Jung and Levin 1999; Jung *et al.* 2002). In *A. fumigatus*, the CWI also relies on the MAPK cascade module of the CWI pathway (Valiante *et al.* 2008, 2009; Dirr *et al.* 2010). To understand how *A. fumigatus* connects the upcoming cell wall damage-derived signal to the downstream cell effectors, we identified and characterized the putative *A. fumigatus RLM1* homolog, *rlmA*, and assigned its function in cell signaling and virulence. The *rlmA* deletion mutant exhibits reduced radial growth and delayed hyphal elongation at different temperatures. These phenotypes indicate defects in the CWI pathway in a similar way to those reported for other CWI pathway mutants (Valiante *et al.* 2008, 2009; Dichtl *et al.* 2012; Samantaray *et al.* 2013; Rocha *et al.* 2015). Indeed, the Δ*rlmA* mutant incorporates phenotypes that are the hallmarks of cell wall perturbation including increased susceptibility to cell wall disturbing agents such as CR, CFW, CAFF echinocandins and nikkomycin Z (an inhibitor of chitin synthase), as previously reported (Damveld *et al.* 2005; Fujioka *et al.* 2007; Levin 2011; Kovacs *et al.* 2013). In addition, *A. fumigatus* RlmA was also able to bind to the MADS-Box motif, one of the main hallmarks of the *RLM1* transcription factor (Damveld *et al.* 2005; Levin 2011), activating luciferase expression. Recently, an independent Δ*rlmA* strain was described and the authors interestingly assigned the role of *rlmA* in the production of (DHN)-melanin by regulating *pksP* (polyketide synthase gene) expression (Valiante *et al.* 2016). In this report, deletion of *rlmA* also resulted in a higher sensitivity to CR compared to the wild-type strain. The Δ*rlmA* mutant also showed impaired resistance to the ER-stressing agents DTT, BFA, and TM. The *A. fumigatus pkcA*^G579R^ mutant also exhibited altered tolerance to these agents and an unbalanced accumulation of the spliced (induced) *hacA* transcript (Rocha *et al.* 2015). These data support the idea that the cell wall biogenesis that occurs during cell wall stress is accompanied by the activation of the Unfolded Protein Response (Malavazi *et al.* 2014; Rocha *et al.* 2015). In considering the range of the cell wall disturbing agents tested here, these results suggest that a lack of *rlmA* leads to alterations in the cell wall structure and/or organization likely interfering with all major polysaccharide moieties that comprise the *A. fumigatus* cell wall. Nevertheless, we cannot infer whether the alkali-soluble or alkali-insoluble fractions of the cell wall are quantitatively altered in this mutant at the moment. However, we indirectly analyzed the chitin and β-glucan contents in Δ*rlmA* germlings via CFW and dectin-1 staining. The mutant presented increased detection of β-glucan and chitin in addition to increased protoplast production when the hyphae were subjected to enzymatic digestion. All these phenotypes are accompanied by a thicker cell wall structure inspected by TEM. In fact, sensitivity to cell wall perturbing agents and carbohydrate compositions are barely correlated in some cases. As we previously noted, the differences in sugar compositions between *A. fumigatus* and other model fungal organisms or fungal pathogens may account for the differences in the activation of cell wall effectors (Rocha *et al.* 2015). For instance, the deletion of *Candida albicans* Δ/Δ*RLM1* resulted in a significant increase in the chitin content and a reduction in mannans (Delgado-Silva *et al.* 2014). On the other hand, *A. nidulans* cell wall composition in the Δ*mpkA* mutant was comparable to that of the wild-type, even though the Δ*mpkA* mutant showed poor growth and sensitivity to micafungin (Yoshimi *et al.* 2015). These results further illustrate the point that it is critical to study the function of cell wall-related genes in fungi. The dramatic changes to which the cell is subjected when a given gene is lacking is the result of a concomitant global perturbation in different cell signaling pathways that are reciprocally affected, which accounts for the final phenotype. This idea is supported by several reports indicating that the complete absence of a given carbohydrate moiety still leads to viable cells (Klimpel and Goldman 1988; Henry *et al.* 2012; Dichtl *et al.* 2015). Further explanations for this result can be obtained from the genetic analyses of the CWI pathway genes. Strikingly, the phenotypes observed for the Δ*rlmA* and *pkcA*^G579R^ mutants were milder than the phenotypes of the Δ*mpkA* strain with regards to colony morphology and cell wall stress caused by CR, CFW, or caspofungin (Figure S6, A–E). We propose that *pkcA*, *mpkA*, and *rlmA* are epistatic in the CWI pathway for the recovery of cell wall stress induced by these compounds, and that the CWI pathway in *A. fumigatus* resembles that of *S. cerevisiae*. However, the MAPK module of the CWI pathway may perform a broader range of multiple roles and interactions inside the cell that result in the severe vegetative growth defects found in these *A. fumigatus* mutants. Some roles of the *mpkA* gene in *A. fumigatus* biology, aside from the ones it plays in the CWI pathway, were previously shown and experimentally exploited, which supports our hypothesis. These *mpkA* roles include the production of secondary metabolites and siderophores (Valiante *et al.* 2009; Jain *et al.* 2011). In fact, the cross talk that occurs between the CWI pathway and other signaling cascades has been described as functioning under different stress conditions such as cell wall and osmotic stress in different organisms (Munro *et al.* 2007; Fuchs and Mylonakis 2009; Garcia *et al.* 2009, 2015; Ouedraogo *et al.* 2011; Fiedler *et al.* 2014). In *A. fumigatus*, the coordinated function of the CWI and high-osmolarity glycerol (HOG) pathways was recently demonstrated during adaptation to caspofungin stress (Altwasser *et al.* 2015). In addition, the contributions of the MAP kinases MpkC and SakA to the CWI pathway were investigated by determining MpkA phosphorylation during CR-induced stress. The results indicated that MpkA phosphorylation was dependent on MpkC and HogA function (Bruder Nascimento *et al.* 2016).

Another explanation for the Δ*rlmA* cell wall-associated phenotypes that were intermediate to those of the Δ*mpkA* strain is that more than one transcription factor is participating in the activation of the cell wall-related genes, an assumption that is supported by the results shown in Figure 8, Figure 9, and Figure S7. Accordingly, when the cells are stressed-out through cell wall damage, the canonical CWI pathway is not acting exclusively in the maintenance of the cell wall in *A. fumigatus*. In fact, this was also reported in *A. niger*, indicating that RlmA is the main transcription factor required for the protection against CFW, but also that it cooperates with MsnA and CrzA to ensure survival of *A. niger* when challenged with caspofungin (Fiedler *et al.* 2014). Here, we observed that some genes (*fksA*, *agsA*, *agsC*, *gel4*, *chsA*, *chsC*, *chsE*, *chsF*, and *chsG*) apparently lost their transcriptional activation in both Δ*rlmA* and Δ*mpkA* mutant backgrounds. On the other hand, MpkA and RlmA are not required for the expression of *gelA* and *gelC*. In addition, *csmB* expression seems to be positively regulated by *rlmA* and negatively regulated by *mpkA* (Figure 9). Therefore, RlmA could not be the only target of MpkA in *A. fumigatus*. Similar to our results, deletion of the *C. glabrata* Rlm1 displayed modestly impaired growth in the presence of micafungin compared with that of the Δ*slt2*. Additionally, Slt2 may be required for Rlm1 to be fully functional in *C. glabrata* (Miyazaki *et al.* 2010).

Even though other unidentified transcription factors might participate in the activation of cell effectors to synthesize the cell wall, the connection between the Bck1-Mkk2-MpkA MAP kinase module and the downstream RlmA transcription factor is reflected in the reduced phosphorylation of MpkA in the Δ*rlmA* mutant after CR exposure. Our data suggest that RlmA positively regulates MpkA phosphorylation. We can speculate that, under an *rlmA* loss-of-function, the canonical CWI pathway operates in a defective fashion and other signaling cascades may be activated in a compensatory manner to maintain cell viability. As a consequence, MpkA phosphorylation is kept at low levels under these conditions. In addition, a nonidentified phosphatase may be acting and promoting dephosphorylation of MpkA to control the activity of the CWI pathway in the absence of RlmA.

*A. fumigatus* mutants for genes belonging to the CWI pathway have shown different results when assayed for virulence. For example, mutants that are deficient in the MAPK (MpkA) or in the apical Protein Kinase C (PkcA), are not attenuated in terms of virulence (Valiante *et al.* 2008; Rocha *et al.* 2015). By contrast, the *A. fumigatus* MAPKK *mkk2* is required for full virulence (Dirr *et al.* 2010) and no information is currently available for the virulence properties of the MAPKKK *bck1*. Our results also indicate that the defective CWI pathway in the Δ*rlmA* mutant is associated with a loss of virulence in the neutropenic murine model of invasive pulmonary aspergillosis. In *Cryptococcus neoformans*, a systematic functional profiling of all transcription factors indicated that most of the studied phenotypes for the Δ*RLM1* mutant were comparable to that of the wild-type strain, including cell wall stress parameters and virulence, which were not attenuated in either an insect or a mouse model (Jung *et al.* 2015). By contrast, the RLM1 deletion mutant in *C. albicans* showed virulence attenuation (Delgado-Silva *et al.* 2014). The Δ*rlmA* mutant strain was also able to stimulate the BMDM to increase secretion of TNF-α, which is one of the major inflammatory mediators that respond to fungal hyphae. This can be explained by the increased exposure of β-glucan in the Δ*rlmA* mutant, which is a potent stimulator of TNF-α release (Hohl *et al.* 2005; Steele *et al.* 2005; Huang *et al.* 2009). The given modifications in the cell wall carbohydrates and proteins observed in the mutant can also contribute to the increased phagocytosis index by the BMDM that was observed here.

In conclusion, the genetic analysis of a null mutant of *rlmA* in *A. fumigatus* strongly suggests that this gene is part of the cell armamentarium that is required to protect the cell wall. In addition, *rlmA* was shown to be involved in the oxidative damage tolerance. The *rlmA* loss-of-function thus directly impacts the virulence of this fungal pathogen. Therefore, an enhanced understanding of the global mechanisms governing the cell wall biogenesis control that emerges from the *A. fumigatus* CWI pathway is of paramount importance, and may impact on the establishment of new strategies for drug discovery targeting to other components required for cell wall reinforcement.

## Supplementary Material

Supplemental Material
