# Supplementary material for: Aspergillus fumigatus MADS-Box Transcription Factor rlmA Is Required for Regulation of the Cell Wall Integrity and Virulence
Source: G3 (Bethesda). 2016 Jul 28;6(9):2983–3002. doi: 10.1534/g3.116.031112 (PMC5015955; doi:10.1534/g3.116.031112)
Supplement: Supplemental Material [file supp_6_9_2983__index.html]

Aspergillus fumigatus MADS-Box Transcription Factor rlmA Is Required for Regulation of the Cell Wall Integrity and Virulence — Supplemental Material 

# *Aspergillus fumigatus* MADS-Box Transcription Factor *rlmA* Is Required for Regulation of the Cell Wall Integrity and Virulence

## Supplemental Material for Rocha *et al.*, 2016

**Files in this Data Supplement:**

- Figure S1 - Generation of the Δ*rlmA* mutant, complemented and RlmA::GFP strain. (.pdf, 2 MB)
- Table S3 - Real-time PCR primers used in this study. (.pdf, 61 KB)
- Figure S2 - Construction of the *PagsA::mulc* cassette. (.pdf, 59 KB)
- Figure S3 - Growth phenotypes of the Δ*rlmA* mutant strain in the presence of CR and CFW. (.pdf, 629 KB)
- Figure S4 - The *A. fumigatus* Δ*rlmA* strain is more sensitive to endoplasmic reticulum (ER)-stressing agents. (.pdf, 51 MB)
- Figure S5 - The Δ*rlmA* mutant has increased sensitivity to the antifungal triazole drugs. (.pdf, 309 KB)
- Figure S6 - Genetic analysis of the CWI pathway single and double mutants. (.pdf, 3 MB)
- Figure S7 - Analysis of the transcriptional responses of wild-type, Δ*rlmA* and Δ*mpkA* mutant strains to CR-induced cell wall stress. (.pdf, 526 KB)
- Table S1 - *A. fumigatus* strains used in this study. (.pdf, 66 KB)
- Table S2 - Primers used in this study for construction of mutant strains. (.pdf, 59 KB)
