# Supplementary material for: Aspergillus fumigatus MADS-Box Transcription Factor rlmA Is Required for Regulation of the Cell Wall Integrity and Virulence
Source: G3 (Bethesda). 2016 Jul 28;6(9):2983–3002. doi: 10.1534/g3.116.031112 (PMC5015955; doi:10.1534/g3.116.031112)
Supplement: Supplemental Material [file supp_g3.116.031112_FigureS1.pdf]

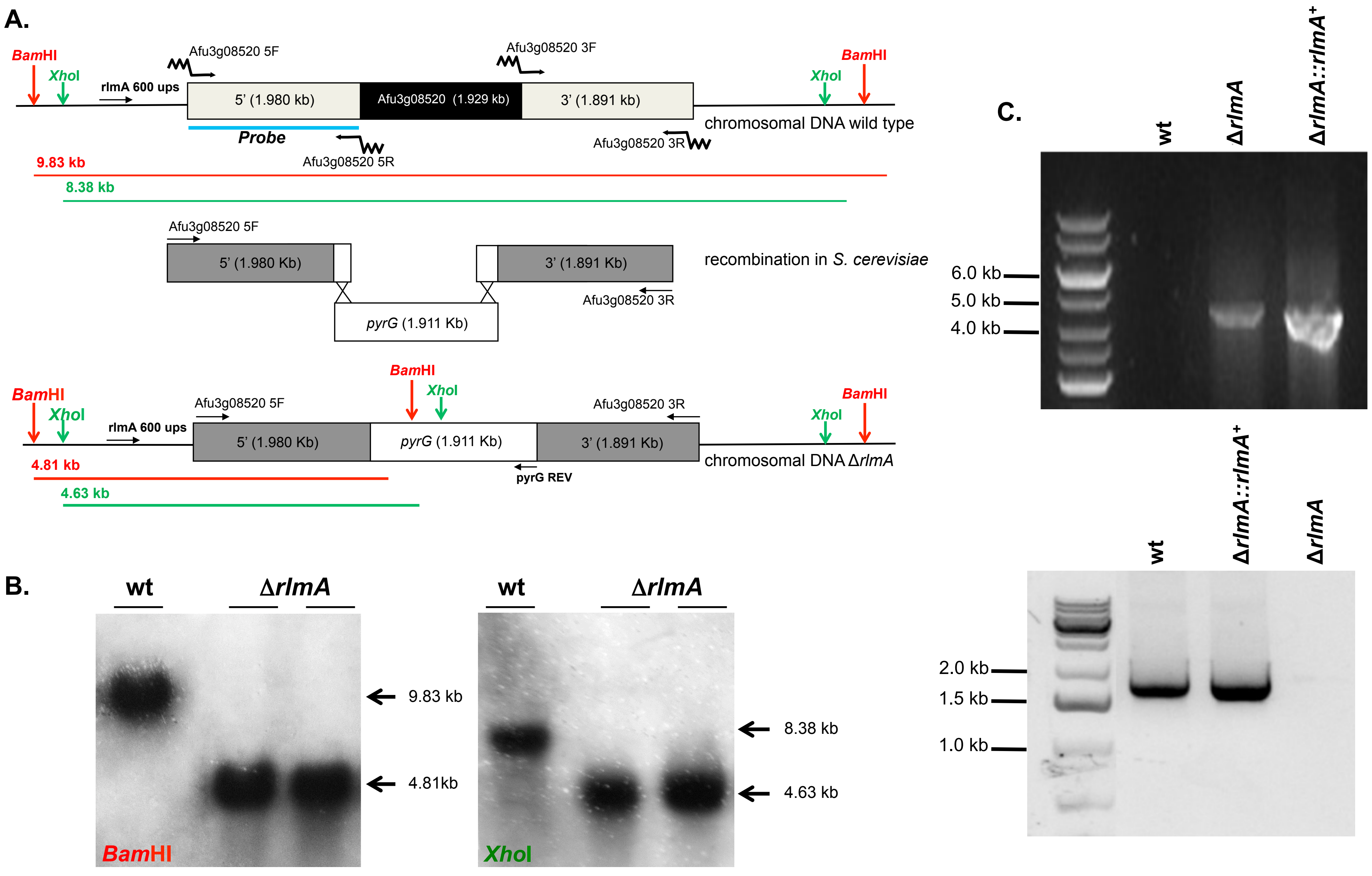

A.
BamHI
XhoI
rlmA 600 ups
Afu3g08520 5F
Afu3g08520 3F
5' (1.980 kb)
Afu3g08520 (1.929 kb)
3' (1.891 kb)
chromosomal DNA wild type
Probe
Afu3g08520 5R
Afu3g08520 3R
9.83 kb
8.38 kb
recombination in S. cerevisiae
5' (1.980 Kb)
3' (1.891 Kb)
pyrG (1.911 Kb)
pyrG REV
chromosomal DNA ΔrlmA
4.81 kb
4.63 kb
B.
wt
ΔrlmA
9.83 kb
4.81kb
8.38 kb
4.63 kb
C.
ΔrlmA::rlmA+
6.0 kb
5.0 kb
4.0 kb
2.0 kb
1.5 kb
1.0 kb

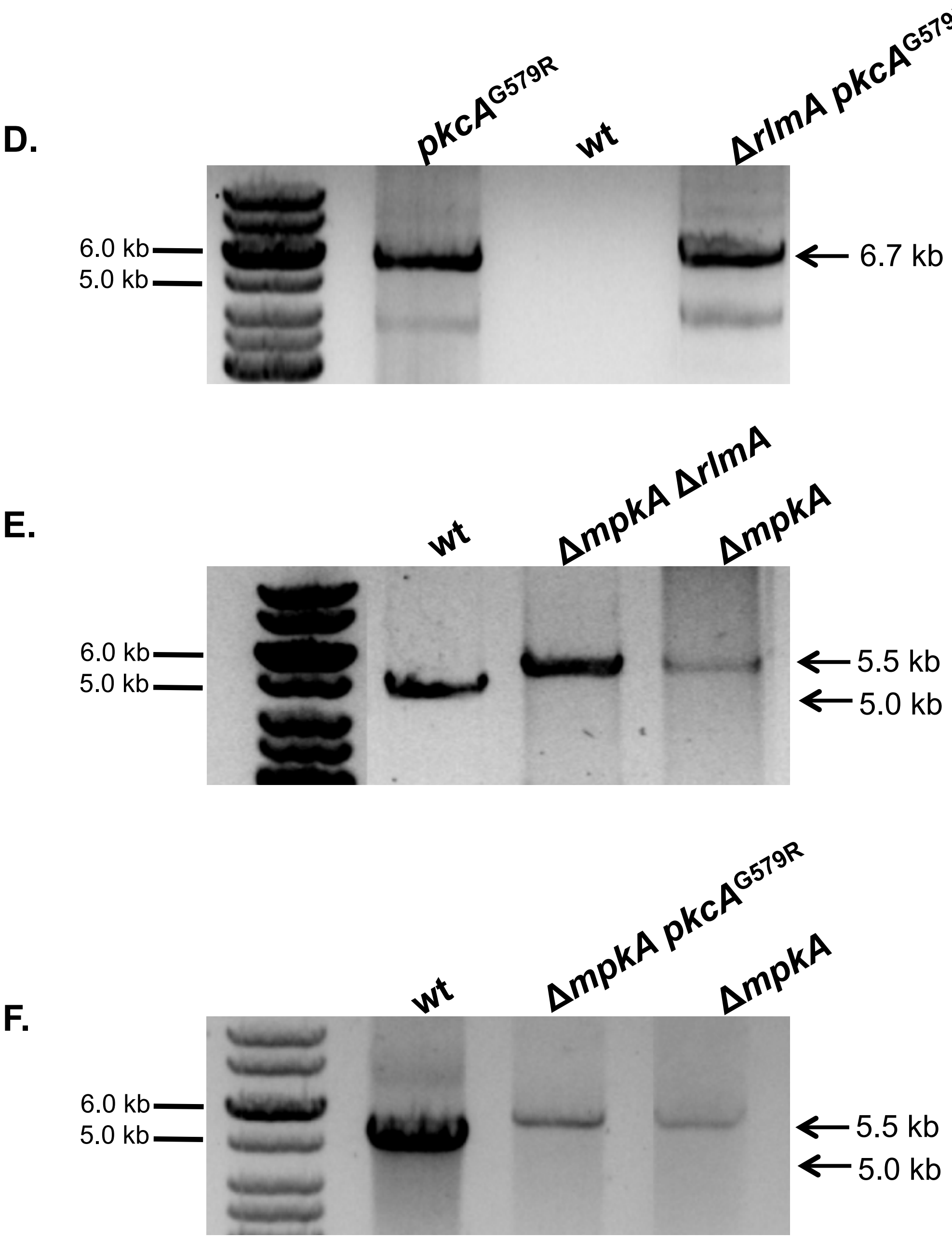
D.
pkcA G579R
wt
ΔrlmA pkcA G579R
6.0 kb
5.0 kb
6.7 kb
E.
wt
ΔmpkA ΔrlmA
ΔmpkA
6.0 kb
5.0 kb
5.5 kb
5.0 kb
F.
wt
ΔmpkA pkcA G579R
ΔmpkA
6.0 kb
5.0 kb
5.5 kb
5.0 kb

G.

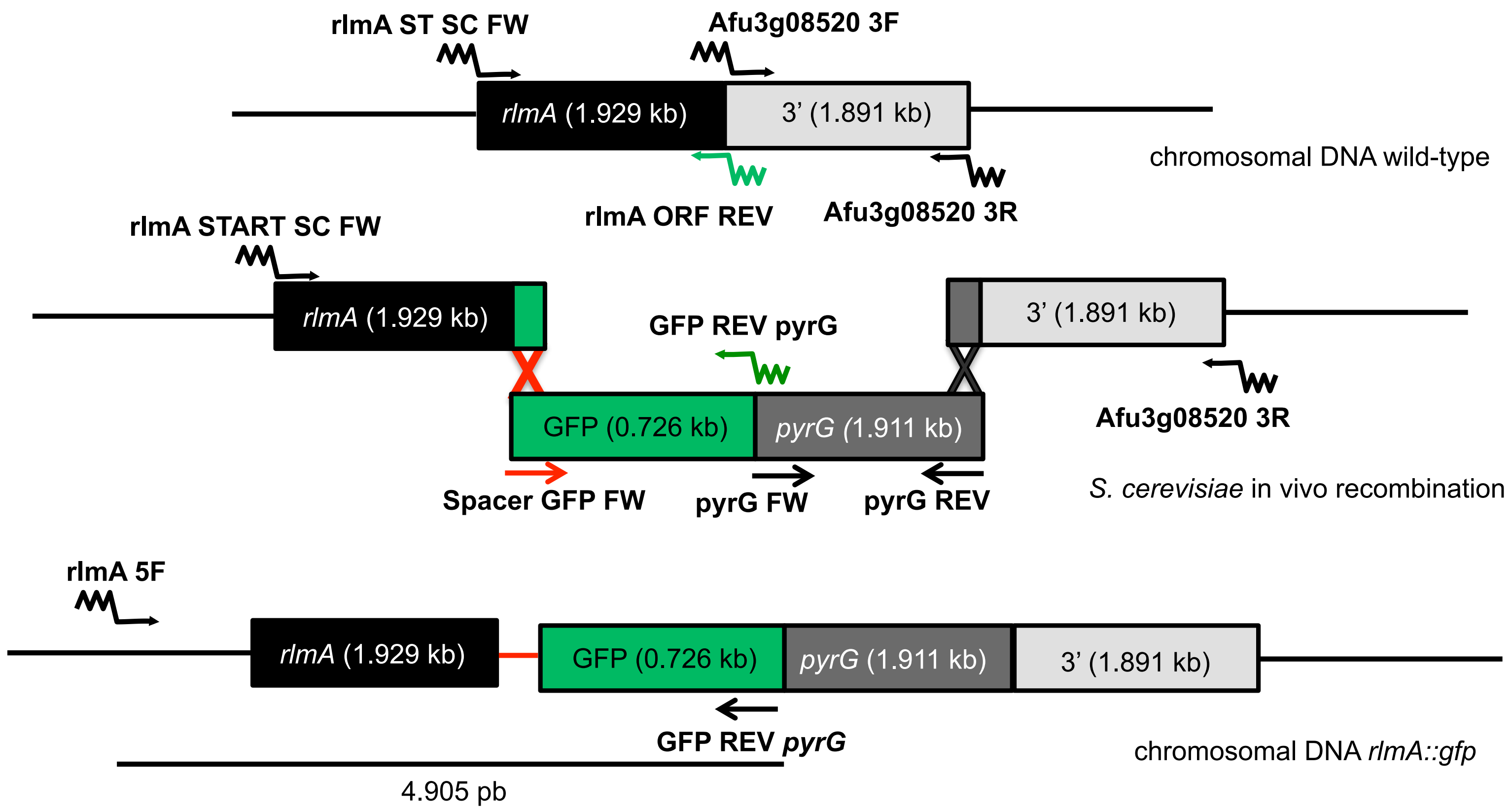
rlmA ST SC FW
Afu3g08520 3F
rlmA (1.929 kb)
3' (1.891 kb)
chromosomal DNA wild-type
rlmA ORF REV
Afu3g08520 3R
rlmA START SC FW
rlmA (1.929 kb)
GFP REV pyrG
3' (1.891 kb)
GFP (0.726 kb)
pyrG (1.911 kb)
Afu3g08520 3R
Spacer GFP FW
pyrG FW
pyrG REV
S. cerevisiae in vivo recombination
rlmA 5F
rlmA (1.929 kb)
GFP (0.726 kb)
pyrG (1.911 kb)
3' (1.891 kb)
GFP REV pyrG
chromosomal DNA rlmA::gfp
4.905 pb

H.

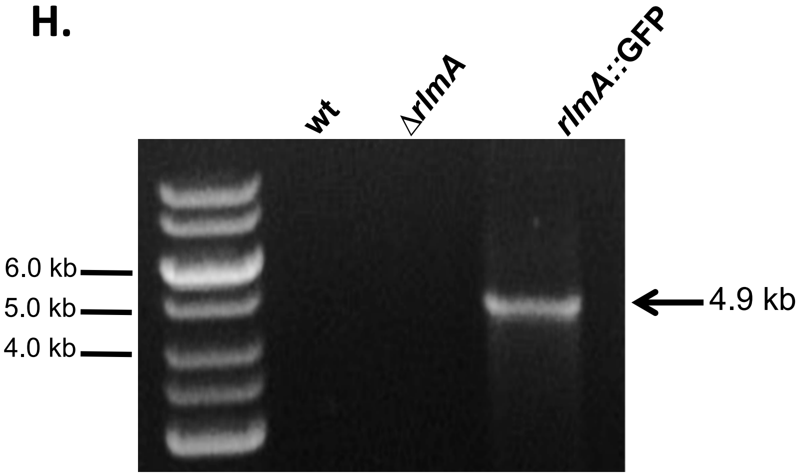
wt
ΔrlmA
rlmA::GFP
6.0 kb
5.0 kb
4.0 kb
4.9 kb

Figure S1 Generation of the Δ*rlmA* mutant, complemented and RlmA::GFP strain. (A) The gene replacement strategy for the *rlmA* gene. The *pyrG* gene was used as a selection marker. The primer names and binding sites are indicated by arrows (see Table S2 for the primer sequences). The deletion cassette was constructed by *in vivo* recombination in *S. cerevisiae*. (B) A Southern blot analysis of *Bam*HI- and *Xho*I-digested genomic DNA with a probe that binds specifically to the *rlmA* 5'-region indicating the predicted 4.81 and 4.63 Kb bands in the Δ*rlmA* mutant for *Bam*HI and *Xho*I DNA digestion, respectively. (C) The successful complementation of the *rlmA* gene was confirmed in CFW- and CR-resistant monoconidial transformants by PCR using primer sets rlmA 600 ups / pyrG REV and rlmA ST SC 5F / rlmA ORF REV (upper and lower panel). (D) PCR for the validation of the CWI pathway double mutants. See text for the details and primers. (G) Gene replacement strategy for *rlmA::gfp* strain construction. The *rlmA* genomic sequence with no stop codon was cloned in-frame with the green fluorescent protein (GFP) gene in a C-terminal fusion separated by a Gly-Thr-Arg-Gly linker. The *pyrG* gene was also used as a prototrophy marker. *S. cerevisiae in vivo* recombination assay was performed as previously described. The PCR-amplified rlmA::gfp cassette was transformed into the *A. fumigatus* wild-type strain. Transformants were carefully tested by PCR with primer set rlmA 5F and GFP REV pyrG to confirm the *rlmA* locus replacement.
