# Supplementary material for: Aspergillus fumigatus MADS-Box Transcription Factor rlmA Is Required for Regulation of the Cell Wall Integrity and Virulence
Source: G3 (Bethesda). 2016 Jul 28;6(9):2983–3002. doi: 10.1534/g3.116.031112 (PMC5015955; doi:10.1534/g3.116.031112)
Supplement: Supplemental Material [file supp_g3.116.031112_TableS3.pdf]

Supplemental Table 3: Real-time PCR primers used in this study

Gene	Systematic name	Primer name	Sequence
pkcA	Afu5g11970	pkcA 2663 FW	5'-CCGAAGTTCTGTTGGCTCTC-3'
		pkcA 2783 REV	5'-CAGAGACCGTAATCGGCAAT-3'
mpkA	Afu4g13720	mpkA FW	5'-GGCCATCAAGAAGGTTACCA-3'
		mpkA REV	5'-TGAAATTGTCTGGTCGTGGA-3'
rlmA	Afu3g08520	rlmA FW'	5'-GACGCCGATCTCTGCTCTAC-3
		rlmA REV	5'-GGAGTGGGGAAGGTTAGAGG-3'
fksA	Afu6g12400	fksA FW	5'-AAGCAATCGAAGCTCAGGAA-3'
		fksA REV	5'-ACCAATCCCATAGAGCGAAC-3'
agsA	Afu3g00910	agsA FW	5'-CCAACACCTGGAAGATGACC-3'
		agsA REV	5'-AACACCGACCGATAGAAGGA-3'
agsB	Afu2g11270	agsB FW	5'-TCAGGGATTGGGCTGTATGT-3'
		agsB REV	5'-TAGCACTTGAGAAGCCAGCA-3'
agsC	Afu1g15440	agsC FW	5'-TGCAGACCCTGACAAGAGTG-3'
		agsC REV	5'-GAACAAGGCAATCCAGAACC-3'
gelA	Afu2g01170	gelA FW	5'-CACTGGCTACGGTCTTCCTC-3'
		gelA REV	5'-CATTGTTGCCGCTAATCTCC-3'
gelB	Afu6g11390	gelB FW	5'-CAGGAGGAGAACGACTACGG-3'
		gelB REV	5'-AGGTCTGGGTTGTGTTGGAG-3'
gelC	Afu2g12850	gelC FW	5'-GAATGGTGCGGTGACAAGAC-3'
		gelC REV	5'-TGTTGCAGCCGTATTCAGAG-3'
gel4	Afu2g05340	gel4 FW	5'-CCTCTGGCCAATGTTGACAA-3'
		gel4 REV	5'-GGTGCGCAGCTGCTTCA-3'
chsA	Afu2g01870	chsA FW	5'-CTGGAGTGTGGCTGGTCTCT-3'
		cshA REV	5'-GCGTGTGAAAGCAGTATGGA-3'
chsB	Afu4g04180	chsB FW	5'-GCTCTCCACTGTCGGTCTCT-3'
		chsB REV	5'-GGTCGTTGTTGATGGTGTTG-3'
chsC	Afu5g00760	chsC FW	5'-TTGCTGCGAGTTTGTATTCC-3'
		chsC REV	5'-GCCAGTAGGATGCCAAAGAG-3'
chsD	Afu1g12600	chsD FW	5'-CAGAACACGATCCGAACAAC-3'
		chsD REV	5'-GCTTCGCACCCAAGTAGAAC-3'
chsE	Afu2g13440	chsE FW	5'-TGGTGTTCGTTGACTTGCTC-3'
		chsE REV	5'-TCATCCATCCAACCATTTCC-3'
chsF	Afu8g05630	chsF FW	5'-AACCTGCTTCTTCTGGGTGA-3'
		chsF REV	5'-GAGCACGAGTTCCATGAGGT-3'
chsG	Afu3g14420	chsG FW	5'-AGGATGAGGGCAAAGAGGTT-3'
		chsG REV	5'-AAGGCGTTGCTAAAGATCCA-3'
csmB	Afu2g13430	csmB FW	5'- ACAATACGCGGCGAATCC-3'
		csmB REV	5'- GTTATCCCGACTGCCCAAAA -3'
sod1	Afu5g09240	sod1 FW	5'- CAAGATCACCGGCACTGTCA-3'
		sod1 REV	5'- AGACGGTGGTGGGAGAGTTCT-3'
sod2	Afu4g11580	sod2 FW	5'-GCTTCGGCTGCTCCAAGA-3'
		sod REV	5'-CCTTGCCGCGAGCAAA-3'
cat2	Afu8g01670	cat2 FW	5'-ACATTGCCGCGCTCAAG-3'
		cat2 REV	5'-GCGGTGGAGATGAAGCTTCT-3'
cat1	Afu3g02270	cat1 FW	5'-TCGGCCCCTGCAGATTC-3'
		cat1 REV	5'-AGCGCCCCAACAGTCTTG-3'
yapA	Afu6g09930	yapA FW	5'-GGAAGCCCATCCCACAATT-3'
		yapA REV	5'-TCTTTGGCGCTGCTGGTT-3'
tubA	Afu1g10910	tubA FW	5'-TTCCCAACAACATCCAGACC-3'
		tubA REV	5'-CGACGGAACATAGCAGTGAA-3'
