# Supplementary material for: Aspergillus fumigatus MADS-Box Transcription Factor rlmA Is Required for Regulation of the Cell Wall Integrity and Virulence
Source: G3 (Bethesda). 2016 Jul 28;6(9):2983–3002. doi: 10.1534/g3.116.031112 (PMC5015955; doi:10.1534/g3.116.031112)
Supplement: Supplemental Material [file supp_g3.116.031112_FigureS2.pdf]

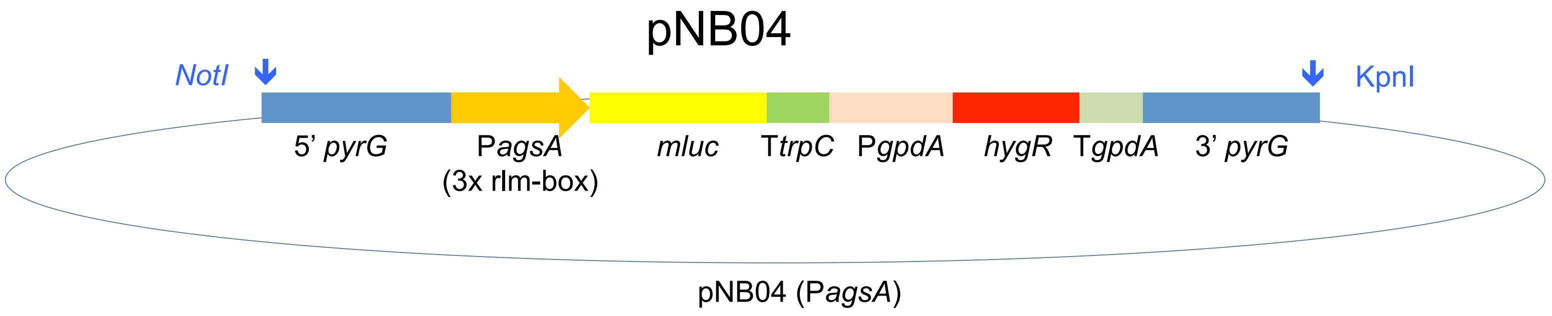

Figure S2 Construction of the *PagsA::mulc* cassette. The plasmid pNB04 contains the sequence of *A. niger agsA* promoter region including 3x rlm recognition domain cloned downstream the luciferase (*mluc*) reporter gene. Hygromicin resistance gene was used as dominant marker and is surrounded by *the A. nidulans gpdA* promoter and termination region. The plasmid contains the 5' and 3'end of *A. fumigatus pyrG* gene to drive homologous integration at the *pyrG* locus. The details on the construction of the plasmid will be published elsewhere.
