# Supplementary material for: Aspergillus fumigatus MADS-Box Transcription Factor rlmA Is Required for Regulation of the Cell Wall Integrity and Virulence
Source: G3 (Bethesda). 2016 Jul 28;6(9):2983–3002. doi: 10.1534/g3.116.031112 (PMC5015955; doi:10.1534/g3.116.031112)
Supplement: Supplemental Material [file supp_g3.116.031112_FigureS3.pdf]

A.

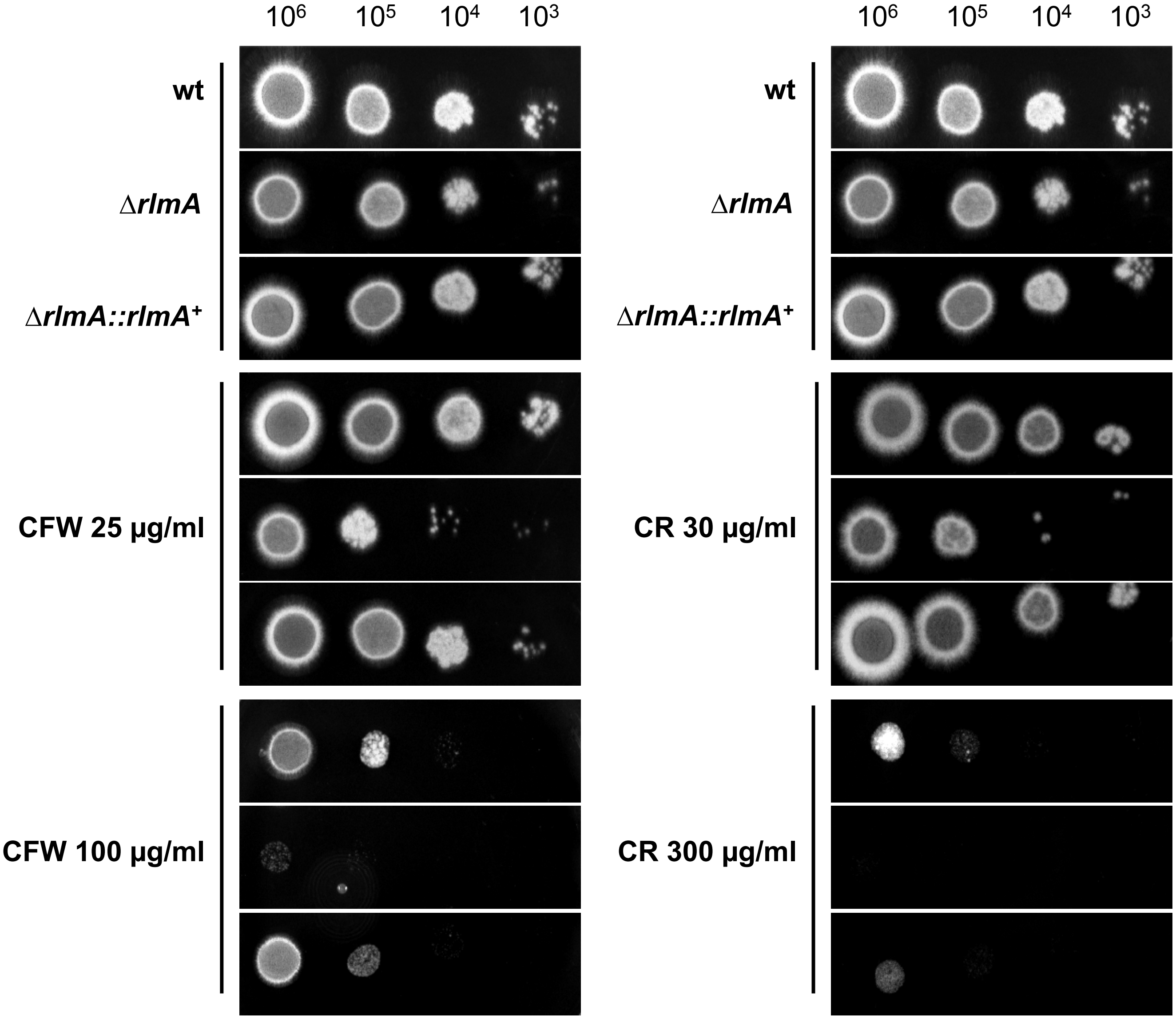
10^6
10^5
10^4
10^3
wt
ΔrlmA
ΔrlmA::rlmA+
CFW 25 µg/ml
CFW 100 µg/ml
10^6
10^5
10^4
10^3
wt
ΔrlmA
ΔrlmA::rlmA+
CR 30 µg/ml
CR 300 µg/ml

B.

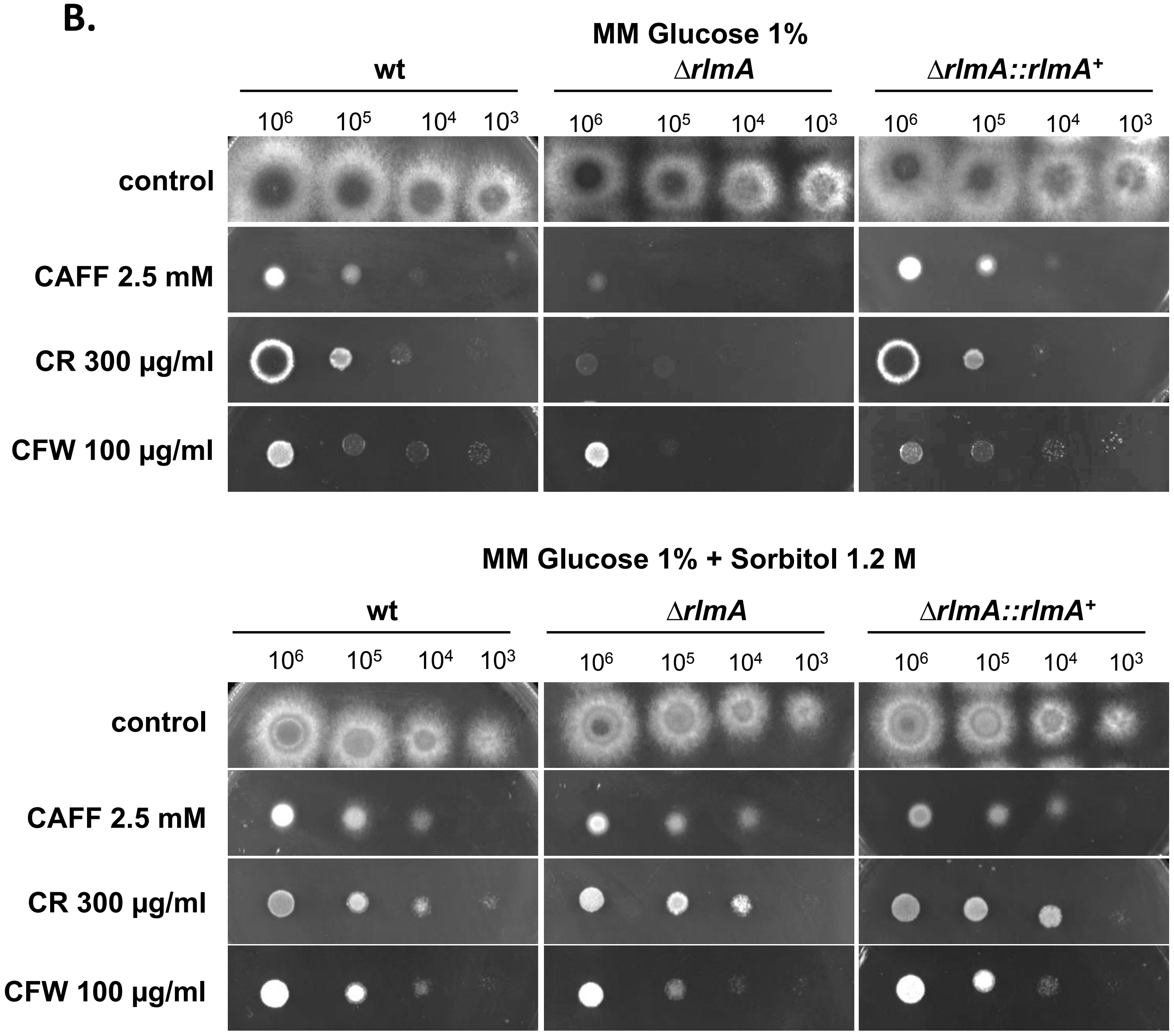

Figure S3 Growth phenotypes of the Δ*rlmA* mutant strain in the presence of CR and CFW. (A) The indicated number of conidia was inoculated into solid YG medium that was supplemented with CR and CFW. The plates were incubated at 37ºC for 72 hours and photographed (A). Growth phenotypes of the Δ*rlmA* mutant in the presence of the osmotic stabilizer D-sorbitol shows a recovery in the sensitivity to cell wall damaging agents. The indicated number of conidia was spotted onto solid MM at 37ºC, with or without 1.2 M of sorbitol supplemented with Caffeine (CAFF), Congo Red (CR) and Calcofluor White (CFW). The plates were incubated for 72 hours and photographed (B).
