# Supplementary material for: Aspergillus fumigatus MADS-Box Transcription Factor rlmA Is Required for Regulation of the Cell Wall Integrity and Virulence
Source: G3 (Bethesda). 2016 Jul 28;6(9):2983–3002. doi: 10.1534/g3.116.031112 (PMC5015955; doi:10.1534/g3.116.031112)
Supplement: Supplemental Material [file supp_g3.116.031112_FigureS4.pdf]

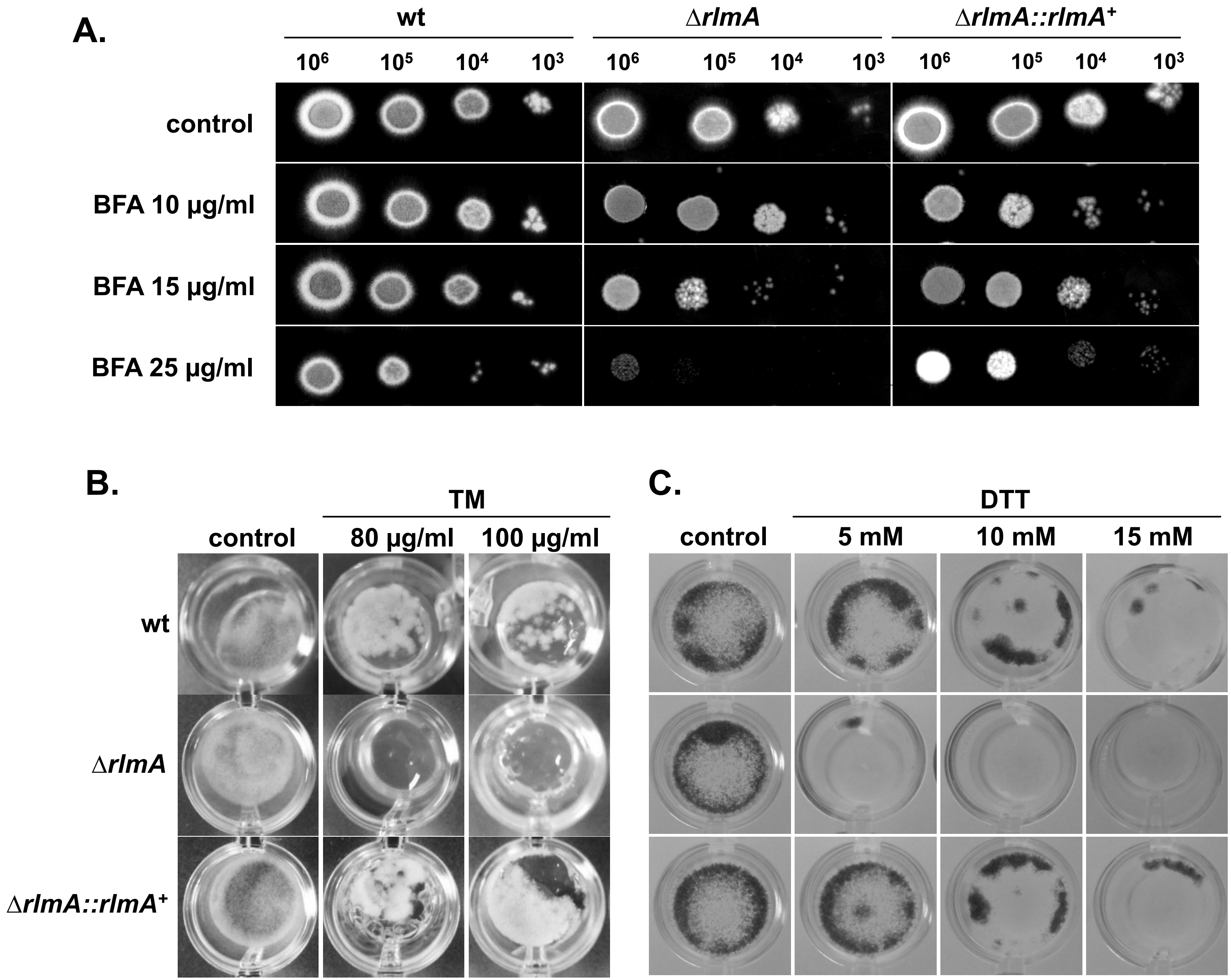
A.
wt
ΔrlmA
ΔrlmA::rlmA+
10^6 10^5 10^4 10^3
control
BFA 10 µg/ml
BFA 15 µg/ml
BFA 25 µg/ml
B.
TM
control
80 µg/ml
100 µg/ml
wt
ΔrlmA
ΔrlmA::rlmA+
C.
DTT
control
5 mM
10 mM
15 mM

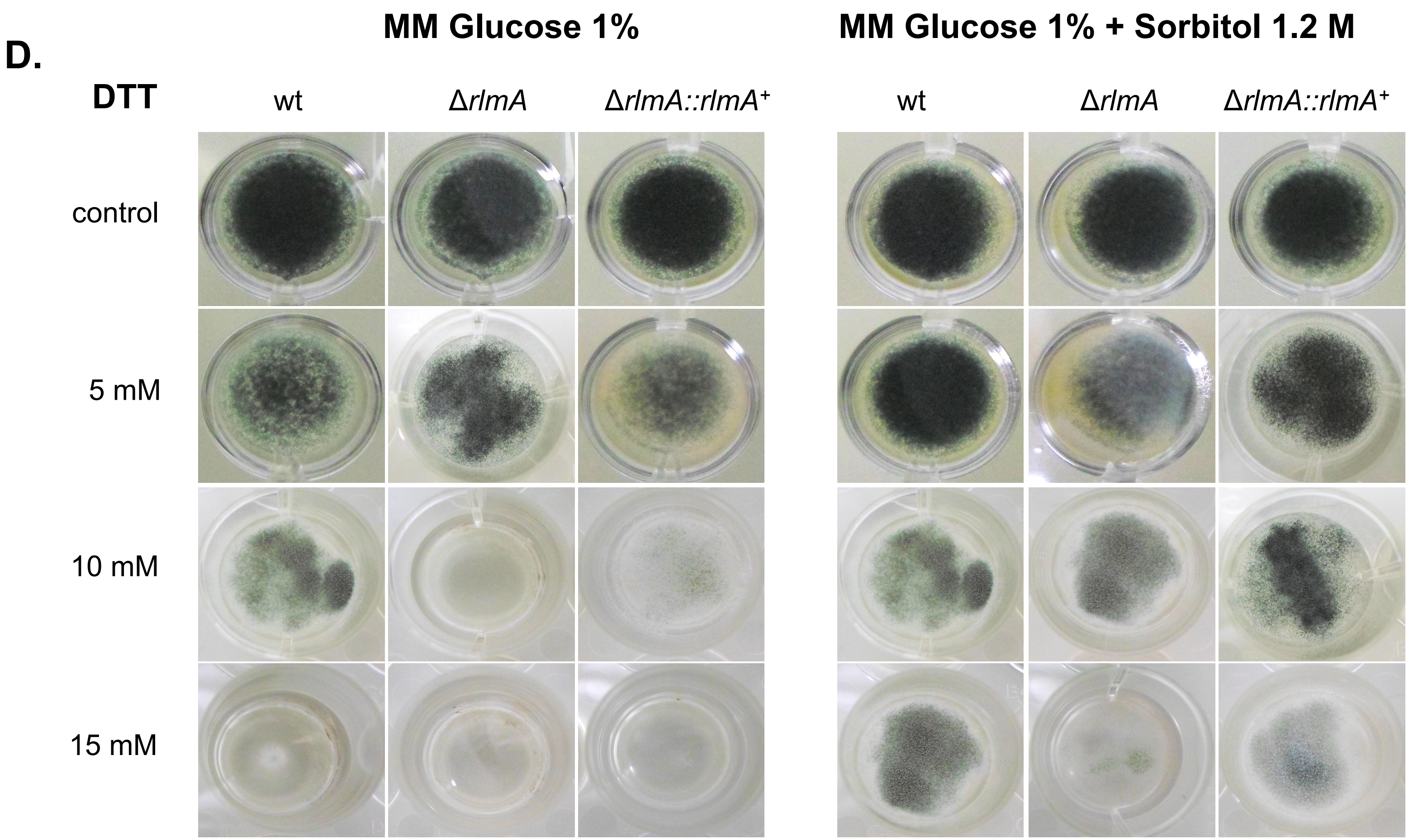


**Figure S4** The *A. fumigatus* Δ*rlmA* strain is more sensitive to endoplasmic reticulum (ER)-stressing agents. The indicated number of conidia from the *A. fumigatus* wild-type, Δ*rlmA* and Δ*rlmA::rlmA*+ strains were spotted on solid YG medium supplemented with brefeldin A (BFA) or into a 24-well plate (1x$10^4$ conidia/well) containing the indicated concentration of tunicamycin (TM) and incubated at 37ºC for 72 hours (A-B). 1x$10^4$ conidia/well were inoculated on liquid YG containing the indicated concentration or dithiothreitol (DTT) and incubated at 37ºC for 48 hours (C). DDT sensitivity of the Δ*rlmA* mutant strain can be rescued by adding D-sorbitol. 1x$10^4$ conidia from the wild-type, Δ*rlmA* and Δ*rlmA::rlmA*$^+$ complemented strain were inoculated into 1 ml of liquid MM in a 24-well plate with or without 1.2 M of sorbitol supplemented with different DTT concentrations. The plates were incubated for 72 hours and photographed (D).
