# Supplementary material for: Aspergillus fumigatus MADS-Box Transcription Factor rlmA Is Required for Regulation of the Cell Wall Integrity and Virulence
Source: G3 (Bethesda). 2016 Jul 28;6(9):2983–3002. doi: 10.1534/g3.116.031112 (PMC5015955; doi:10.1534/g3.116.031112)
Supplement: Supplemental Material [file supp_g3.116.031112_FigureS5.pdf]

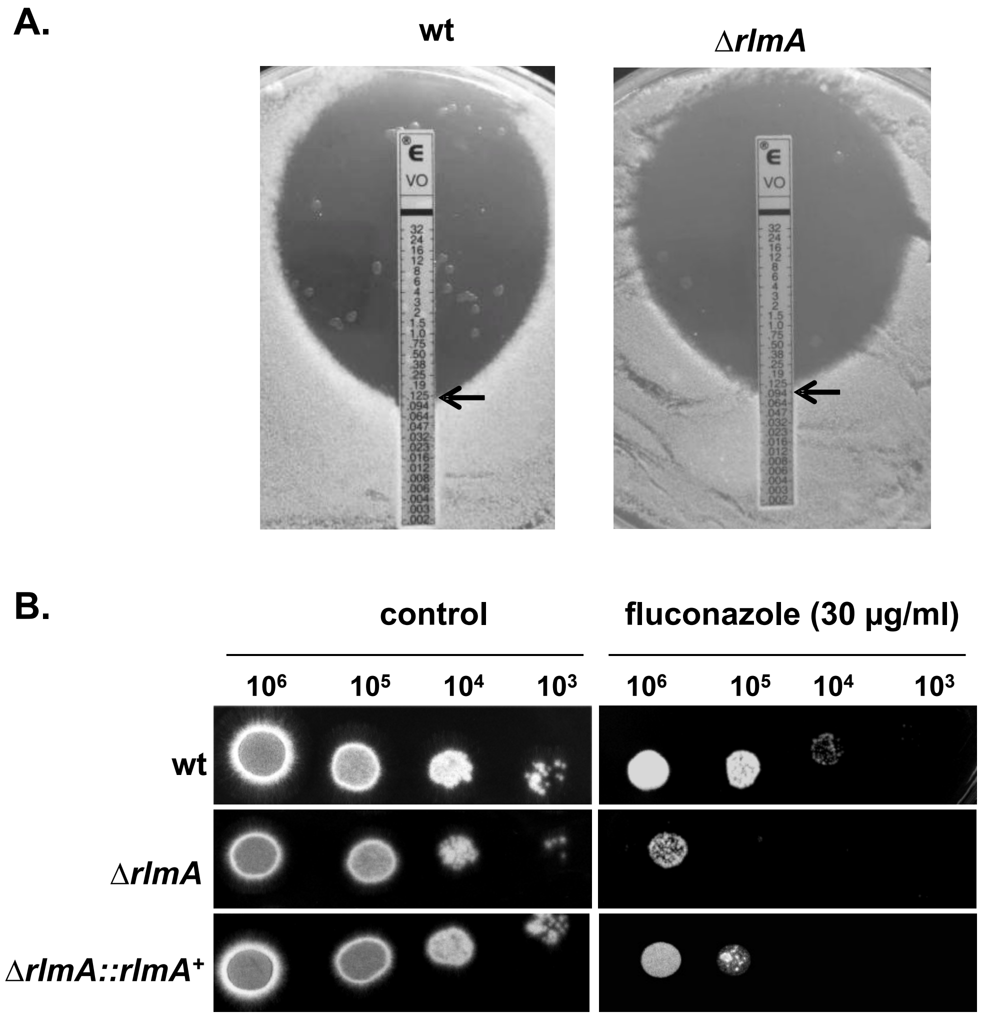

Figure S5 The Δ*rlmA* mutant has increased sensitivity to the antifungal triazole drugs. (A) The antifungal susceptibility according to E-test gradient strips for voriconazole. Endpoints 0.125 (wild-type) and 0.094 (Δ*rlmA*). (B) The indicated numbers of conidia were inoculated onto solid YG plates that were supplemented with fluconazole. The plates were incubated for 48 hours at 37ºC.
