# Supplementary material for: Aspergillus fumigatus MADS-Box Transcription Factor rlmA Is Required for Regulation of the Cell Wall Integrity and Virulence
Source: G3 (Bethesda). 2016 Jul 28;6(9):2983–3002. doi: 10.1534/g3.116.031112 (PMC5015955; doi:10.1534/g3.116.031112)
Supplement: Supplemental Material [file supp_g3.116.031112_FigureS6.pdf]

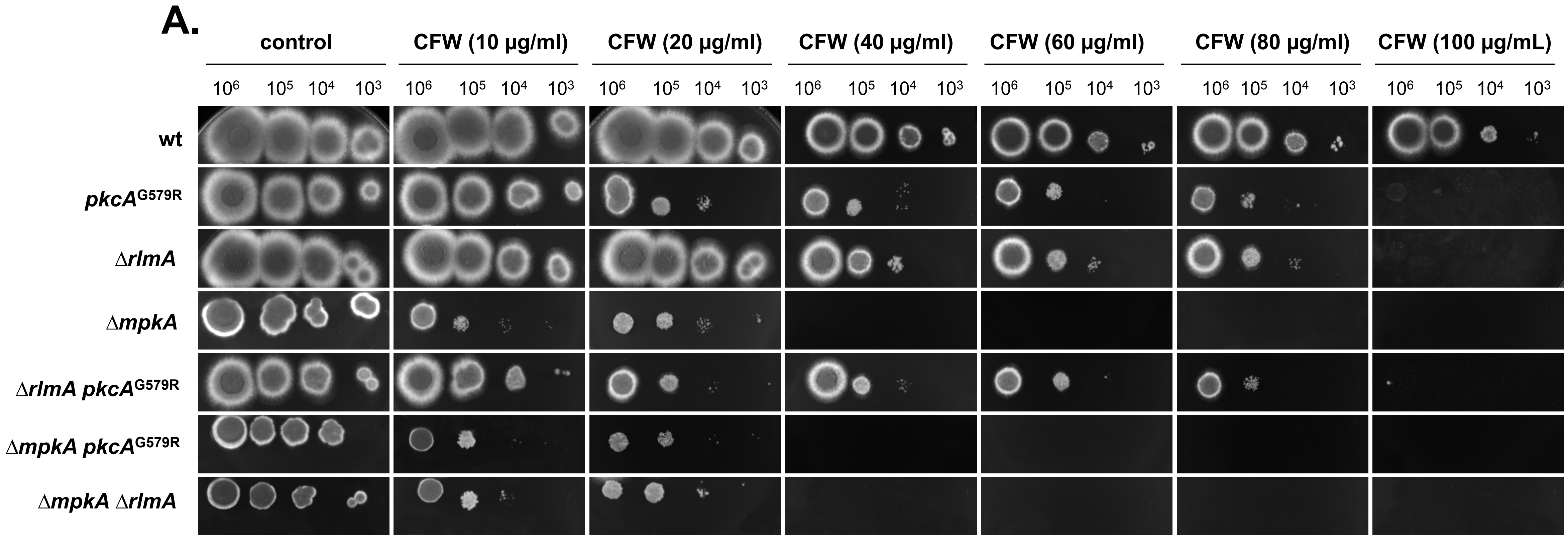
A.
control
CFW (10 µg/ml)
CFW (20 µg/ml)
CFW (40 µg/ml)
CFW (60 µg/ml)
CFW (80 µg/ml)
CFW (100 µg/mL)
10^6 10^5 10^4 10^3
wt
pkcA G579R
ΔrlmA
ΔmpkA
ΔrlmA pkcA G579R
ΔmpkA pkcA G579R
ΔmpkA ΔrlmA

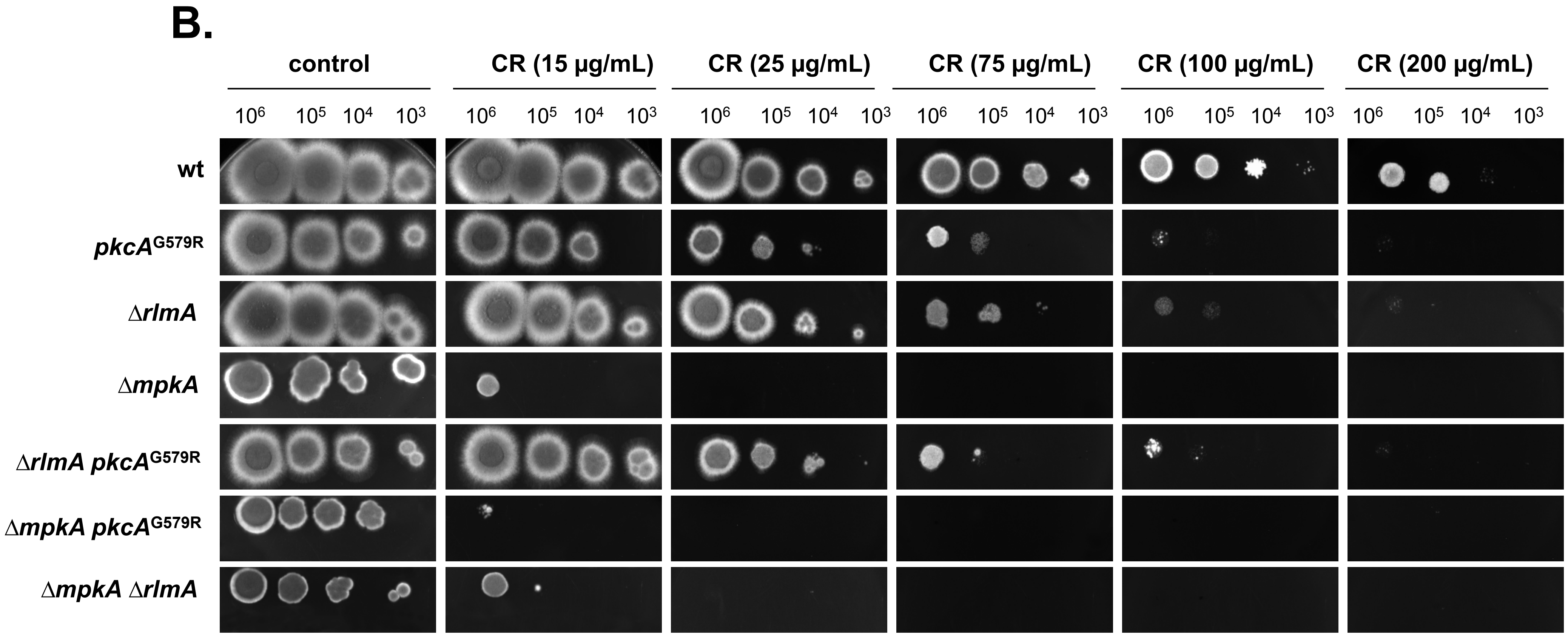

B.
control
CR (15 μg/mL)
CR (25 μg/mL)
CR (75 μg/mL)
CR (100 μg/mL)
CR (200 μg/mL)
10^6 10^5 10^4 10^3
wt
pkcA^G579R
ΔrlmA
ΔmpkA
ΔrlmA pkcA^G579R
ΔmpkA pkcA^G579R
ΔmpkA ΔrlmA

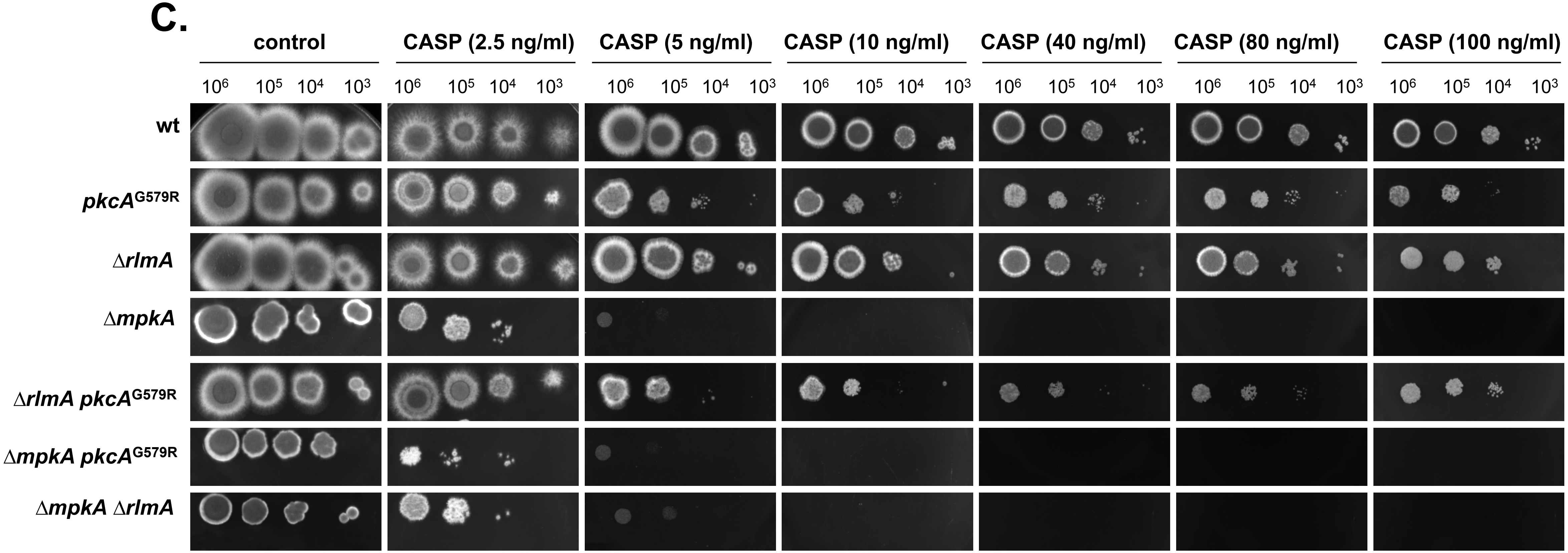
C.
control
CASP (2.5 ng/ml)
CASP (5 ng/ml)
CASP (10 ng/ml)
CASP (40 ng/ml)
CASP (80 ng/ml)
CASP (100 ng/ml)
10⁶ 10⁵ 10⁴ 10³
wt
pkcAG579R
ΔrlmA
ΔmpkA
ΔrlmA pkcAG579R
ΔmpkA pkcAG579R
ΔmpkA ΔrlmA

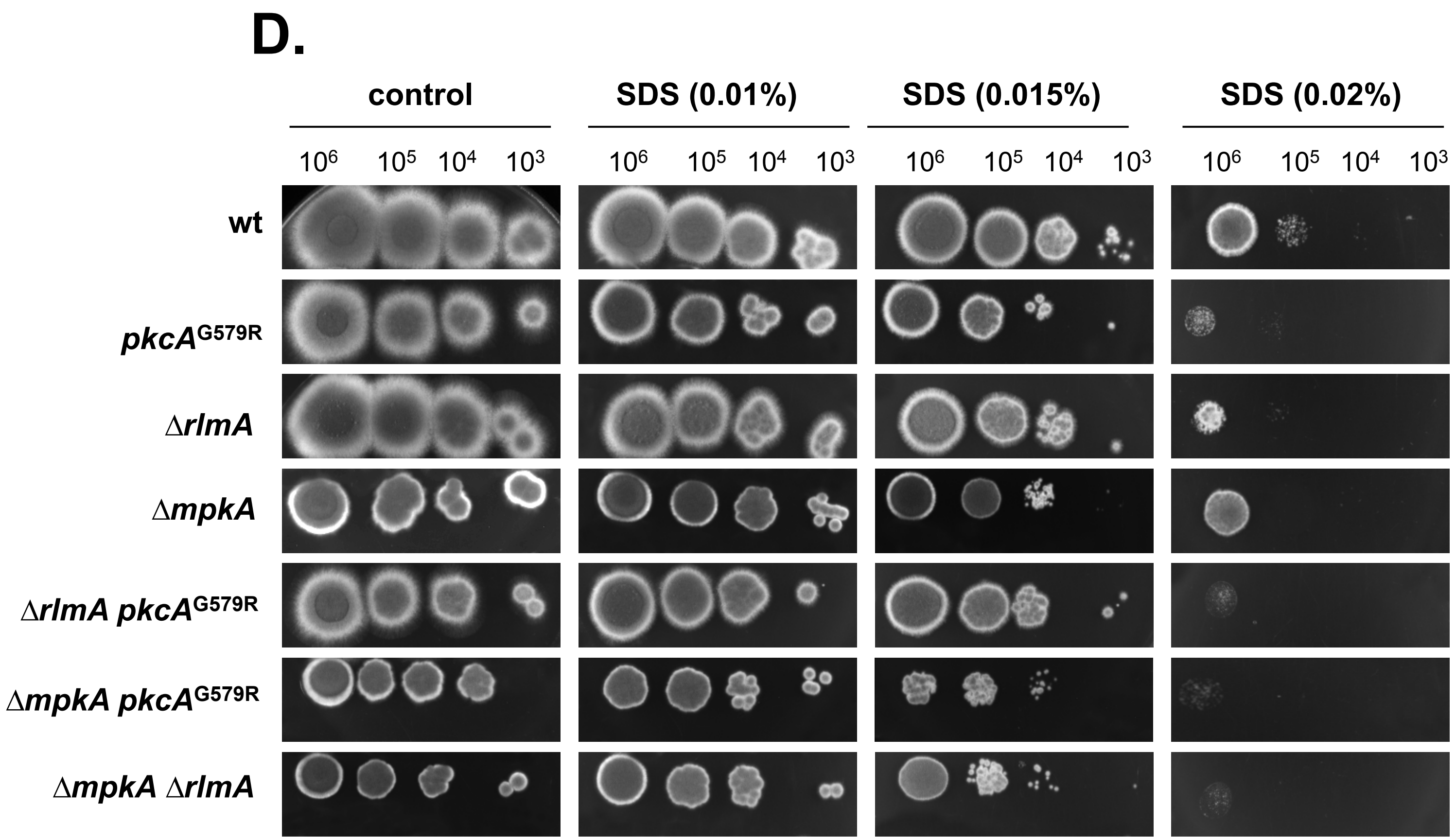

Figure S6 Genetic analysis of the CWI pathway single and double mutants. (A-D) *rlmA*, *pkcA* and *mpkA* interact genetically during cell wall stress in the presence of CFW, CR, caspofungin and SDS.
