# Supplementary material for: Aspergillus fumigatus MADS-Box Transcription Factor rlmA Is Required for Regulation of the Cell Wall Integrity and Virulence
Source: G3 (Bethesda). 2016 Jul 28;6(9):2983–3002. doi: 10.1534/g3.116.031112 (PMC5015955; doi:10.1534/g3.116.031112)
Supplement: Supplemental Material [file supp_g3.116.031112_FigureS7.pdf]

A.

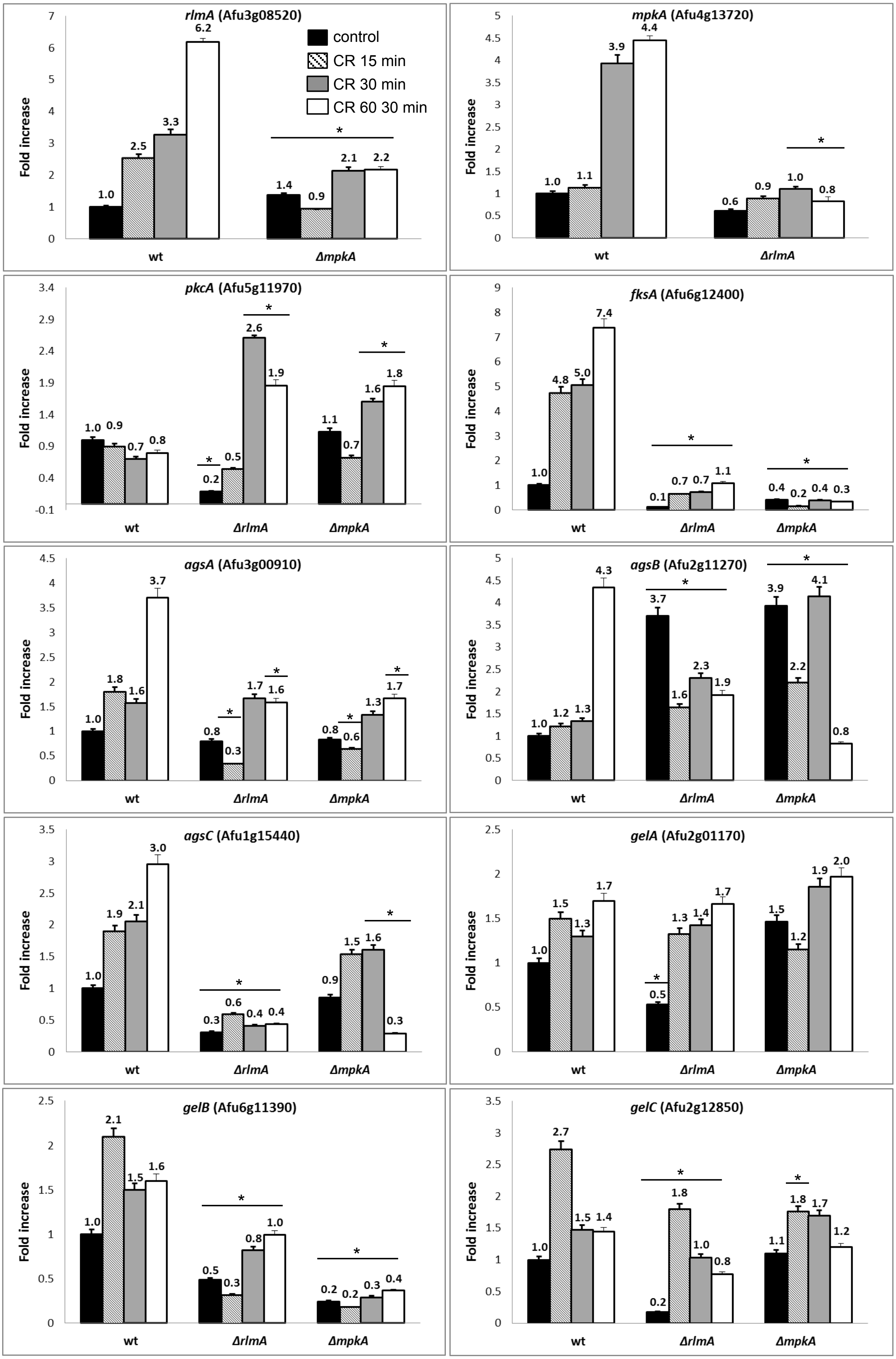
rlmA (Afu3g08520)
control
CR 15 min
CR 30 min
CR 60 30 min
Fold increase
1.0
2.5
3.3
6.2
1.4
0.9
2.1
2.2
*
wt
ΔmpkA
mpkA (Afu4g13720)
1.0
1.1
3.9
4.4
0.6
0.9
1.0
0.8
wt
ΔrlmA
pkcA (Afu5g11970)
1.0
0.9
0.7
0.8
0.2
0.5
2.6
1.9
1.1
0.7
1.6
1.8
wt
ΔrlmA
ΔmpkA
fksA (Afu6g12400)
1.0
4.8
5.0
7.4
0.1
0.7
0.7
1.1
0.4
0.2
0.4
0.3
agsA (Afu3g00910)
1.0
1.8
1.6
3.7
0.8
0.3
1.7
1.6
0.8
0.6
1.3
1.7
agsB (Afu2g11270)
1.0
1.2
1.3
4.3
3.7
1.6
2.3
1.9
3.9
2.2
4.1
0.8
agsC (Afu1g15440)
1.0
1.9
2.1
3.0
0.3
0.6
0.4
0.4
0.9
1.5
1.6
0.3
gelA (Afu2g01170)
1.0
1.5
1.3
1.7
0.5
1.3
1.4
1.7
1.5
1.2
1.9
2.0
gelB (Afu6g11390)
1.0
2.1
1.5
1.6
0.5
0.3
0.8
1.0
0.2
0.2
0.3
0.4
gelC (Afu2g12850)
1.0
2.7
1.5
1.4
0.2
1.8
1.0
0.8
1.1
1.8
1.7
1.2

B.

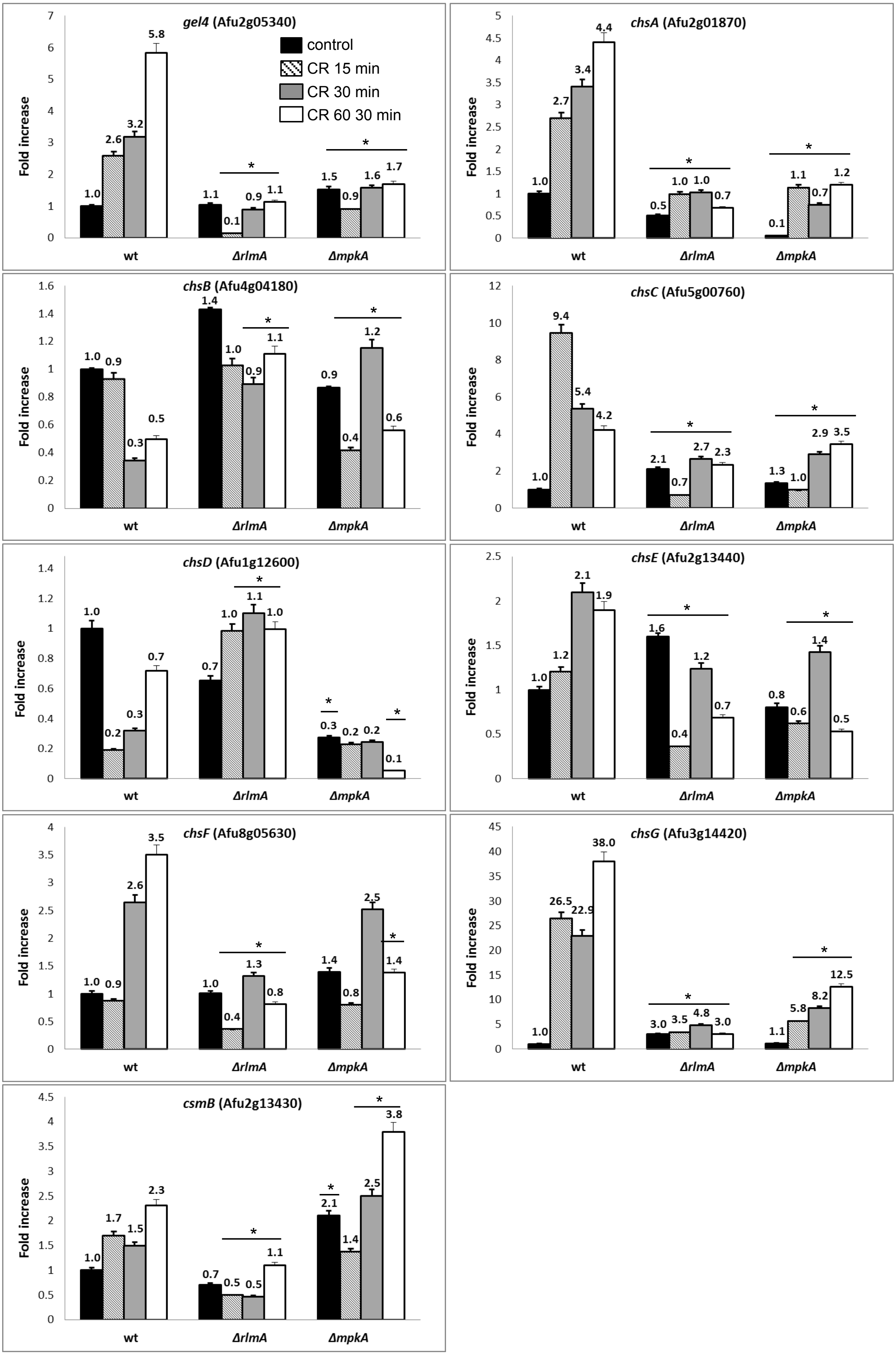
gel4 (Afu2g05340)
control
CR 15 min
CR 30 min
CR 60 30 min
Fold increase
1.0 2.6 3.2 5.8
1.1 0.1 0.9 1.1
1.5 0.9 1.6 1.7
*
wt
ΔrlmA
ΔmpkA
chsA (Afu2g01870)
1.0 2.7 3.4 4.4
0.5 1.0 1.0 0.7
0.1 1.1 0.7 1.2
chsB (Afu4g04180)
1.0 0.9 0.3 0.5
1.4 1.0 0.9 1.1
0.9 0.4 1.2 0.6
chsC (Afu5g00760)
1.0 9.4 5.4 4.2
2.1 0.7 2.7 2.3
1.3 1.0 2.9 3.5
chsD (Afu1g12600)
1.0 0.2 0.3 0.7
0.7 1.0 1.1 1.0
0.3 0.2 0.2 0.1
chsE (Afu2g13440)
1.0 1.2 2.1 1.9
1.6 0.4 1.2 0.7
0.8 0.6 1.4 0.5
chsF (Afu8g05630)
1.0 0.9 2.6 3.5
1.0 0.4 1.3 0.8
1.4 0.8 2.5 1.4
chsG (Afu3g14420)
1.0 26.5 22.9 38.0
3.0 3.5 4.8 3.0
1.1 5.8 8.2 12.5
csmB (Afu2g13430)
1.0 1.7 1.5 2.3
0.7 0.5 0.5 1.1
2.1 1.4 2.5 3.8

Figure S7 Analysis of the transcriptional responses of wild-type, Δ*rlmA* and Δ*mpkA* mutant strains to CR-induced cell wall stress. The wild-type, Δ*rlmA* and Δ*mpkA* strains were grown in liquid MM medium. After incubation, CR was added and the cultures and grown for an additional 15, 30 and 60 min. The mRNA abundance for each gene was assessed by qRT-PCR and normalized to β-tubulin (A-B). The fold increase in each strain represents the normalized mRNA abundance relative to the wild-type strain at time point 0 (i.e., prior to CR exposure). The data represent the average value of at least three biological replicates, each of which was repeated in duplicate in the same run, Bar = SD, * $p \leq 0.05$ (One Way ANOVA).
