# Supplementary material for: Aspergillus fumigatus MADS-Box Transcription Factor rlmA Is Required for Regulation of the Cell Wall Integrity and Virulence
Source: G3 (Bethesda). 2016 Jul 28;6(9):2983–3002. doi: 10.1534/g3.116.031112 (PMC5015955; doi:10.1534/g3.116.031112)
Supplement: Supplemental Material [file supp_g3.116.031112_TableS1.pdf]

Supplemental Table 1: *A. fumigatus* strains used in this study.

Name	Genotype	Reference
akuB^{KU80}	Δ*akuB; pyrG-*	da Silva Ferreira *et al.*, 2006
Δ*rlmA*	Δ*rlmA::pyrG;* Δ*akuB*	This study
Δ*rlmA::rlmA*$^{+}$	Δ*rlmA::rlmA*$^{+}$*;* Δ*akuB*	This study
Δ*rlmA pyrG-*	Δ*rlmA;* Δ*akuB*	This study
rlmA::gfp	*rlmA::gfp;* Δ*akuB*	This study
pkcA^{G579R}	*pkcA*^{G579R}*::pyrG;* Δ*akuB*	Rocha *et al.*, 2015
Δ*mpkA*	Δ*mpkA;* Δ*akuB*	Valiante *et al.*, 2009
Δ*rlmA; pkcA*^{G579R}	Δ*rlmA; pkcA*^{G579R}*;* Δ*akuB*	This study
Δ*rlmA;* Δ*mpkA*	Δ*rlmA;* Δ*mpkA;* Δ*akuB*	This study
pkcA^{G579R}*;* Δ*mpkA*	*pkcA*^{G579R}*;* Δ*mpkA*	This study
MAF 6.6	P*agsA*::*mluc;* Δ*akuB*	This study
MAF 8.1	Δ*rlmA*; P*agsA*::*mluc;* Δ*akuB*	This study

References

da Silva Ferreira, M.E., Kress, M.R., Savoldi, M., Goldman, M.H., Hartl, A., Heinekamp, T., *et al.*, (2006) The akuB(KU80) mutant deficient for nonhomologous end joining is a powerful tool for analyzing pathogenicity in Aspergillus fumigatus. *Eukaryot Cell* **5**: 207-211.

Rocha, M.C., Godoy, K.F., de Castro, P.A., Hori, J.I., Bom, V.L., Brown, N.A., *et al.*, (2015) The Aspergillus fumigatus pkcAG579R Mutant Is Defective in the Activation of the Cell Wall Integrity Pathway but Is Dispensable for Virulence in a Neutropenic Mouse Infection Model. *PLoS One* **10**: e0135195.

Valiante, V., Jain, R., Heinekamp, T. and Brakhage, A.A., (2009) The MpkA MAP kinase module regulates cell wall integrity signaling and pyomelanin formation in Aspergillus fumigatus. *Fungal Genet Biol* **46**: 909-918.
