# Supplementary material for: Aspergillus fumigatus MADS-Box Transcription Factor rlmA Is Required for Regulation of the Cell Wall Integrity and Virulence
Source: G3 (Bethesda). 2016 Jul 28;6(9):2983–3002. doi: 10.1534/g3.116.031112 (PMC5015955; doi:10.1534/g3.116.031112)
Supplement: Supplemental Material [file supp_g3.116.031112_TableS2.pdf]

Supplemental Table 2: Primers used in this study for construction of mutant strains.

Primer name*	Sequence
Afu3g08520 5F	5’-gtaacgccagggttttcccagtcacgacgCTTGCTTGCTTGCTTGCTT-3’
Afu3g08520 5R	5’-gcatcagtgcctcctctcagacagaattccGCAGTAAACAAGGAACACCACG-3’
pyrG FW	5’-GGAATTCTGTCTGAGAGGAGGC-3’
pyrG REV	5’-GATATCGAATTCGCCTCAAAC-3’
Afu3g0852 3F	5’-aagagcattgtttgaggcgaattcgatatcACTTTCTTGAATATTGCGATGG-3’
Afu3g0852 3R	5’-gcggataacaatttcacacaggaaacagcTCAGCGTCCGGTAAGTTG-3’
rlmA 1000 FW	5’-CGTGTGGTTGCCAGCCTTGACCAGG-3’
rlmA 1000 REV	5’-TCGTCTACCAGAACGACCTCGCGCG-3’
rlmA ST SC 5F	5’ – gtaacgccagggttttcccagtcacgacgATGGGTCGAAGAAAGATCGA-3’
rlmA ORF REV	5’-agttcttctcctttactcattccccgtgttccCGTCTTGGATTTCTTCGCC-3’
rlmA 600 ups	5’-GAATGAGAAGAAAGGAGGAATGA-3’
Spacer GFP FW	5'-**GGAACACGGGGA**ATGAGTAAAGGAGAAGAACTTTTCA-3’
GFP REV pyrG	**5’-**GCATCAGTGCCTCCTCTCAGACAGAATTCCTTATTTGTATAGTTCATCCATGCCATG-3’
MpkA_5’_For§	5'-CTCATTCCTTGTTCTGATGCG-3'
MpkA_3’_Rev§	5'-GACTGTCGCAGAAATCCGCTT-3'
mpkA 600 ups	5’-GAGCCCTGACTTCACTGCA-3’
cpkcA FW¶	5’-TACAACATACCTGGCTGGATG-3’

Small letters indicate homology to the pRS426 flanking sequence
Small underlined letters indicate homology to a fragment in the cassette
Bold letters indicate the Gly-Thr-Arg-Gly linker separating *rlmA* C-terminus and GFP start codon.
* For primers location refer to Figure S1.
§ Sequence from Valiante *et al.*, 2009
¶ Sequence from Rocha *et al.*, 2015

References

Rocha, M.C., Godoy, K.F., de Castro, P.A., Hori, J.I., Bom, V.L., Brown, N.A., *et al.*, (2015) The Aspergillus fumigatus pkcAG579R Mutant Is Defective in the Activation of the Cell Wall Integrity Pathway but Is Dispensable for Virulence in a Neutropenic Mouse Infection Model. *PLoS One* **10**: e0135195.

Valiante, V., Jain, R., Heinekamp, T. and Brakhage, A.A., (2009) The MpkA MAP kinase module regulates cell wall integrity signaling and pyomelanin formation in Aspergillus fumigatus. *Fungal Genet Biol* **46**: 909-918.
